# Microfungi associated with *Azadirachta* (*Meliaceae*, *Sapindales*): New species, new records, and a global checklist

**DOI:** 10.3897/mycokeys.142.208876

**Published:** 2026-10-14

**Authors:** Ashani D. Madagammana, Chitrabhanu S. Bhunjun, Chayanard Phukhamsakda, Dong-qin Dai, Sajad Ali, Samantha C. Karunarathna, Kevin D. Hyde, Haohan Wang

**Affiliations:** 1 Center for Yunnan Plateau Biological Resources Protection and Utilization, College of Biological Resource and Food Engineering, Qujing Normal University, Qujing 655011, China Center for Yunnan Plateau Biological Resources Protection and Utilization, College of Biological Resource and Food Engineering, Qujing Normal University Qujing China; 2 Center of Excellence in Fungal Research, Mae Fah Luang University, Chiang Rai 57100, Thailand Center of Excellence in Fungal Research, Mae Fah Luang University Chiang Rai Thailand; 3 School of Science, Mae Fah Luang University, Chiang Rai 57100, Thailand School of Science, Mae Fah Luang University Chiang Rai Thailand; 4 Mushroom Research Foundation, 128 M.3 Ban Pa Deng T. Pa Pae, A. Mae Taeng, Chiang Mai 50150, Thailand Mushroom Research Foundation Chiang Mai Thailand; 5 Department Microbial Drugs, Helmholtz Centre for Infection Research (HZI), Inhoffenstrasse 7, 38124, Braunschweig, Germany Department Microbial Drugs, Helmholtz Centre for Infection Research (HZI) Braunschweig Germany; 6 Center for Yunnan Plateau Biological Resources, Protection and Utilization, Key Laboratory of Yunnan Provincial Department of Education of the Deep-Time Evolution on Biodiversity from the Origin of the Pearl River, Qujing Normal University, Qujing, Yunnan Province, 655011, China Center for Yunnan Plateau Biological Resources, Protection and Utilization, Key Laboratory of Yunnan Provincial Department of Education of the Deep-Time Evolution on Biodiversity from the Origin of the Pearl River, Qujing Normal University Qujing China; 7 Department of Biological Sciences, College of Science, King Faisal University, Al-Ahsa 31982, Saudi Arabia Department of Biological Sciences, College of Science, King Faisal University Al-Ahsa Saudi Arabia; 8 National Institute of Fundamental Studies, Hantane Road, Kandy 2000, Sri Lanka National Institute of Fundamental Studies Kandy Sri Lanka; 9 Department of Botany and Microbiology, College of Science, King Saud University, P.O. Box 2455, Riyadh 11451, Saudi Arabia Department of Botany and Microbiology, College of Science, King Saud University Riyadh Saudi Arabia; 10 CAS Key Laboratory for Plant Diversity and Biogeography of East Asia, Kunming Institute of Botany, Chinese Academy of Science, Kunming, Yunnan 650201, China CAS Key Laboratory for Plant Diversity and Biogeography of East Asia, Kunming Institute of Botany, Chinese Academy of Science Kunming China; 11 Innovative Institute for Plant Health, Zhongkai University of Agriculture and Engineering, Guangzhou 510225, China Innovative Institute for Plant Health, Zhongkai University of Agriculture and Engineering Guangzhou China

**Keywords:** Five species, fungal inventory, morphology, multi-gene phylogeny, neem, taxonomy

## Abstract

*Azadirachta (Meliaceae)* is a small genus comprising economically and ecologically important plants, including *A. indica*, which is widely used in traditional medicine and other applications such as natural pest control and cosmetics. Several studies have previously been conducted on *Azadirachta*, including its phytochemistry and associated microfungi, particularly endophytic species inhabiting the *A. indica* host. In this study, dead stems of *Azadirachta indica* bearing fungal fruiting bodies were collected, and the fungi were isolated. Based on morphology and multi-locus (ITS, LSU, SSU, *tef*1-α, and *rpb*2) phylogenetic analyses using maximum likelihood (ML) and Bayesian inference (BI), the taxonomic placements of the isolated fungal strains were determined. Five taxa are reported, including two new species, *Neomassaria azadirachtae* and *Nigrograna azadirachtae*; *Aquadictyospora yunnanensis* as a new host and geographical record; and *Neoaquastroma bauhiniae* and *Pararoussoella lincangensis* as two additional new host records. All new isolates in this study belong to *Pleosporales* (*Dothideomycetes*, *Ascomycota*), contributing to the understanding of the diversity of *Pleosporales* taxa associated with *Azadirachta*. In addition, a global checklist of 212 microfungal taxa and fungus-like *Oomycota* (excluding *Basidiomycota*) reported from *Azadirachta* species has been compiled. Of the two known *Azadirachta* species (*A. indica* and *A. excelsa*), most fungal species were recorded from *A. indica* (210 species), whereas only one was reported from *A. excelsa*. This study provides an updated overview of fungal diversity associated with *Azadirachta*, particularly *A. indica*, with a global checklist and new saprobic collections including new species, host records, and geographical records.

## Introduction

The plant family *Meliaceae*, commonly known as the mahogany family, is currently classified in *Sapindales* and includes over 50 genera (Yadav et al. 2015; Cui et al. 2023; Amusa et al. 2024). *Meliaceae* is characterised by simple, imparipinnate or paripinnate compound leaves, stamens that unite to form a cylindrical tube, and syncarpous, apparently bisexual flowers borne in panicles, cymes, spikes, or clusters (Amusa et al. 2024). However, seed, floral, and fruit morphologies within this family are highly diverse (Amusa et al. 2024). This plant family is important for its high-quality timber, and researchers have extracted several important chemical compounds from some species (Cui et al. 2023).

Within the diverse genera of *Meliaceae*, *Azadirachta* is one of the most important lineages that has received notable attention in scientific studies. *Azadirachta* is a genus of evergreen trees that reach heights of 15–50 m (He et al. 2022). Several studies have explored the biochemical properties, medicinal applications, and different fungal communities associated with *Azadirachta* species (Chutulo and Chalannavar 2018; Saleem et al. 2018). Notably, fungal communities associated with *Azadirachta* have been investigated in several countries, particularly endophytic fungi inhabiting the leaves, stems, bark, and roots (Shao et al. 2008; Verma et al. 2011; Al-Daghari et al. 2020). Additionally, Chutulo and Chalannavar (2018) compiled an extensive account of the endophytic mycoflora of *Azadirachta indica*, highlighting both fungal diversity and their bioactive potential. In Thailand, microfungi associated with *Azadirachta* have received limited attention. A recent study of endophytic fungi included *Azadirachta indica* among Thai medicinal plants examined by Jarussophon et al. (2026). Although these studies have provided valuable insights, a comprehensive, up-to-date global checklist of microfungi associated with *Azadirachta* has not yet been established. Moreover, while endophytic fungi associated with *Azadirachta* have been relatively well studied, their saprobic fungal communities remain understudied. Plant-rich habitats provide a diverse array of litter substrates that support diverse saprobic fungal communities, which play important roles in litter decomposition and nutrient cycling (Queiroz et al. 2021). Investigating these less-studied fungi helps fill a key gap in biodiversity research while supporting taxonomic studies and understanding the flexibility of life modes and distribution patterns in fungal species (Hyde et al. 2024a).

Among microfungi associated with *Azadirachta*, species of *Pleosporales* were the most frequently recorded taxa (Shao et al. 2008; Verma et al. 2011). Barr (1987) formally introduced *Pleosporales*. This is the largest order in *Dothideomycetes*, accounting for one-quarter of all described dothideomycetous species (Hongsanan et al. 2020; Hyde et al. 2020a; Pem et al. 2024; Thiyagaraja et al. 2025). To date, pleosporalean taxa have been reported from various habitats with different lifestyles, including endophytic and pathogenic associations with living leaves and stems; saprobic lifestyles on dead plant stems, leaves, and bark; hyperparasitism on fungi and insects; and lichenised associations (Bhunjun et al. 2021, 2026; Phukhamsakda et al. 2022; Su et al. 2022; Xu et al. 2022). Currently, 91 families and 653 genera are listed under this order in the latest fungal outline by Hyde et al. (2024b). In Thailand, several fungal diversity studies have reported *Pleosporales* as a prominent order with relatively high species representation (Htet et al. 2025; Karimi et al. 2025).

In this study, five saprobic pleosporalean taxa associated with *A. indica* across five families and genera were described and reported. Among these taxa, two new species, *Neomassaria azadirachtae* and *Nigrograna azadirachtae*, are introduced, together with a new host and geographical record of *Aquadictyospora yunnanensis* and two new host records of *Pleopunctum ellipsoideum* and *Neoaquastroma bauhiniae* associated with *A. indica* in northern Thailand. Additionally, an updated global checklist of microfungi associated with *Azadirachta* was compiled, which is important for understanding fungal diversity in this host plant genus. Linking the global checklist of microfungi in *Azadirachta* with newly collected fungal species from this study provides a comprehensive, up-to-date framework for understanding fungal diversity within *Azadirachta*. By incorporating taxonomic novelties and new records, this perspective expands the taxonomic and ecological range of microfungi associated with *Azadirachta* and highlights current knowledge gaps that could inform future biodiversity and ecological studies of this host genus.

## Materials and methods

### Sample collection, isolation, and morphological studies

Dead stems of *Azadirachta indica* plants with saprobic fungal fruiting bodies were collected from June to October 2024 in Chiang Rai, Thailand, and the collection information was recorded according to the instructions of Rathnayaka et al. (2025). Specimens were brought to the laboratory in zip-lock bags and examined using the methods described in Senanayake et al. (2020). External examinations were conducted using an OLYMPUS SZXI6 stereomicroscope (Olympus Corporation, Japan). Micromorphological characteristics of fungi were observed using a Nikon ECLIPSE Ni-U compound microscope (Nikon Corporation, Japan) and photographed with a Nikon DS-Ri2 digital camera fitted to the microscope. Measurements of the micromorphological characters were made with the Tarosoft (R) Image Frame Work program (version 0.9.7), and images were processed with Adobe Photoshop CS5 software (Adobe Systems Inc.). Pure fungal colonies were obtained through single-spore isolation, as described by Senanayake et al. (2020). Germinating spores were transferred aseptically to potato dextrose agar (PDA) medium. The cultures were incubated at 25–30 °C for 4–6 weeks with frequent observations. The specimens were deposited in the Mae Fah Luang University Herbarium (**MFLU**), and cultures were deposited in the Mae Fah Luang University Culture Collection (**MFLUCC**). Faces of Fungi (**FoF**) (https://www.facesoffungi.org/) and Index Fungorum (**IF**) numbers (http://www.indexfungorum.org) were obtained for the new species as described by Jayasiri et al. (2015) and Index Fungorum (2026), respectively. Data were also deposited in the Greater Mekong Subregion database of microfungi (Chaiwan et al. 2021).

### DNA extraction, PCR amplification, and sequencing

Fresh mycelium (50–100 mg) scraped from the margin of colonies on PDA plates incubated at 25–30 °C for 4 weeks was used for DNA extraction. OMEGA DNA extraction kits (Bio-Tek Inc., Georgia, USA) were used according to the manufacturer’s instructions. Polymerase chain reaction (PCR) was carried out in a total volume of 25 µL, including 12.5 µL of 2× Power Taq PCR Master Mix, 1 µL of each primer (20 µM), 1 µL of genomic DNA (50–100 ng), and 9.5 µL of deionised water. The internal transcribed spacer region (ITS), the large subunit rDNA (LSU), the small subunit rDNA (SSU), the partial translation elongation factor 1-α gene (*tef*1-α), and the RNA polymerase II second largest subunit gene (*rpb*2) were amplified using the primers and PCR thermal cycle programs presented in Table 1. The PCR products were visualised on a 1% agarose gel (1 μL/10 mL agarose) and immediately stored at 4 °C until sequencing. PCR products were sequenced by Biogenomed Co., Ltd. (South Korea).

**Table 1.** Loci, primers, and PCR thermal cycle programmes used in this study, with corresponding references.

**Loci**	**PCR primers (forward/reverse)**	**PCR conditions**	**References**
ITS	ITS1/ITS4	(94 °C: 30 s, 56 °C: 30 s, 72 °C: 30 sec)	White et al. (1990)
LSU	LR0R/LR5	(94 °C: 30 s, 56 °C: 30 s, 72 °C: 1 min)	Vilgalys and Hester (1990)
SSU	NS1/NS4	(94 °C: 30 s, 56 °C: 30 s, 72 °C: 1 min)	White et al. (1990)
*tef*1-α	EF1-983/EF1-2218R	(94 °C: 30 s, 52 °C: 30 s, 72 °C: 1 min)	Rehner (2001)
*rpb*2	fRPB2-5F/fRPB2-7cR	(94 °C: 30 s, 52 °C: 30 s, 72 °C: 1 min)	Liu et al. (1999)

N.B.: Initial denaturation at 95 °C for 5 min and final extension at 72 °C for 5 min; the number of PCR thermal cycles for each gene region was 35.

### Phylogenetic analyses

BioEdit version 7.2 was used to assess sequence quality (Hall 1999). Lasergene SeqMan Pro version 7 (DNASTAR, Inc., USA) was used to assemble the sequences. BLASTn searches were performed to identify sequences with high similarity indices (https://blast.ncbi.nlm.nih.gov/Blast.cgi) (Sayers et al. 2019). Reference sequences for phylogenetic analyses were downloaded from the NCBI database. Sequence alignments were performed with MAFFT version 7 (Katoh et al. 2019) and manually edited in BioEdit version 7.2. The gappyout option in TrimAl version 1.2 was used to trim the alignments (Capella-Gutiérrez et al. 2009). Single- and multiple-sequence datasets were analysed separately using maximum likelihood (ML) and Bayesian inference (BI). The ML analyses were performed with RAxML-HPC v.8 on ACCESS via the CIPRES Science Gateway (Miller et al. 2010) with 1,000 pseudoreplicates. jModelTest2 on ACCESS was used to estimate the best-fit evolutionary models for each gene using the Akaike Information Criterion (AIC) (Nylander 2004). The BI analyses were performed using MrBayes on the CIPRES Science Gateway (Miller et al. 2010) with Markov chain Monte Carlo (MCMC) sampling, with trees sampled every 100^th^ generation. Convergence was assessed based on the average standard deviation of split frequencies, with values equal to or below 0.01 considered indicative of convergence. FigTree version 1.4.0 was used to visualise the phylograms (Rambaut 2012), and Microsoft PowerPoint (2021) was used to edit the phylograms. The genetic distances between the new species and their sister taxa were calculated using the Kimura 2-parameter method in MEGA X with the pairwise deletion gap option (Kumar et al. 2018).

### Checklist compilation

An updated global checklist of microfungal taxa and fungus-like *Oomycota* reported from *Azadirachta* species up to 2025 (excluding *Basidiomycota*) was compiled (Suppl. material 1) based on published literature, including research papers, books, and authoritative online databases such as Index Fungorum, GenBank, and the USDA (United States Department of Agriculture) fungal database (Farr et al. 2026; Index Fungorum 2026). Five additional taxa documented in the present study were subsequently incorporated into the checklist. In cases where original publications were inaccessible, information was retrieved from authoritative online databases or later peer-reviewed studies that cited those records. Only information explicitly provided in the consulted source was included. The checklist includes information on life mode, host part (substrate), and recorded country, based solely on records from *Azadirachta*. The checklist was arranged at the species level, with each fungal taxon listed only once, regardless of the number of occurrences, life modes, host parts/substrates, countries, or reported studies. When the same microfungal species was reported on different *Azadirachta* species, each association was listed as a separate record in the checklist. Further, species reported from *Azadirachta* but with limited information (e.g. life modes, host parts/substrates, or recorded country) were nevertheless included in the checklist without inference. Species names reported in the literature were checked against current taxonomic sources and updated to currently accepted names. Furthermore, invalid or unresolvable species names were standardised to the genus level.

### Data analysis

Descriptive analyses were performed to summarise the species composition of microfungi at the class and family levels reported from *Azadirachta indica*, as well as their reported life modes. Species composition at the class and family levels was illustrated using pie charts, while the distribution of reported life modes was illustrated using a bar chart. Species with uncertain taxonomic status or unresolved nomenclatural placements, such as *Chromosporium* and *Botryodiplodia*, were included in the checklist but excluded from family- and order-level summaries.

## Results

### Phylogenetic analyses

Five distinct phylogenetic analyses were conducted using ML and BI tree construction methods in this study.

**Figure 1.**
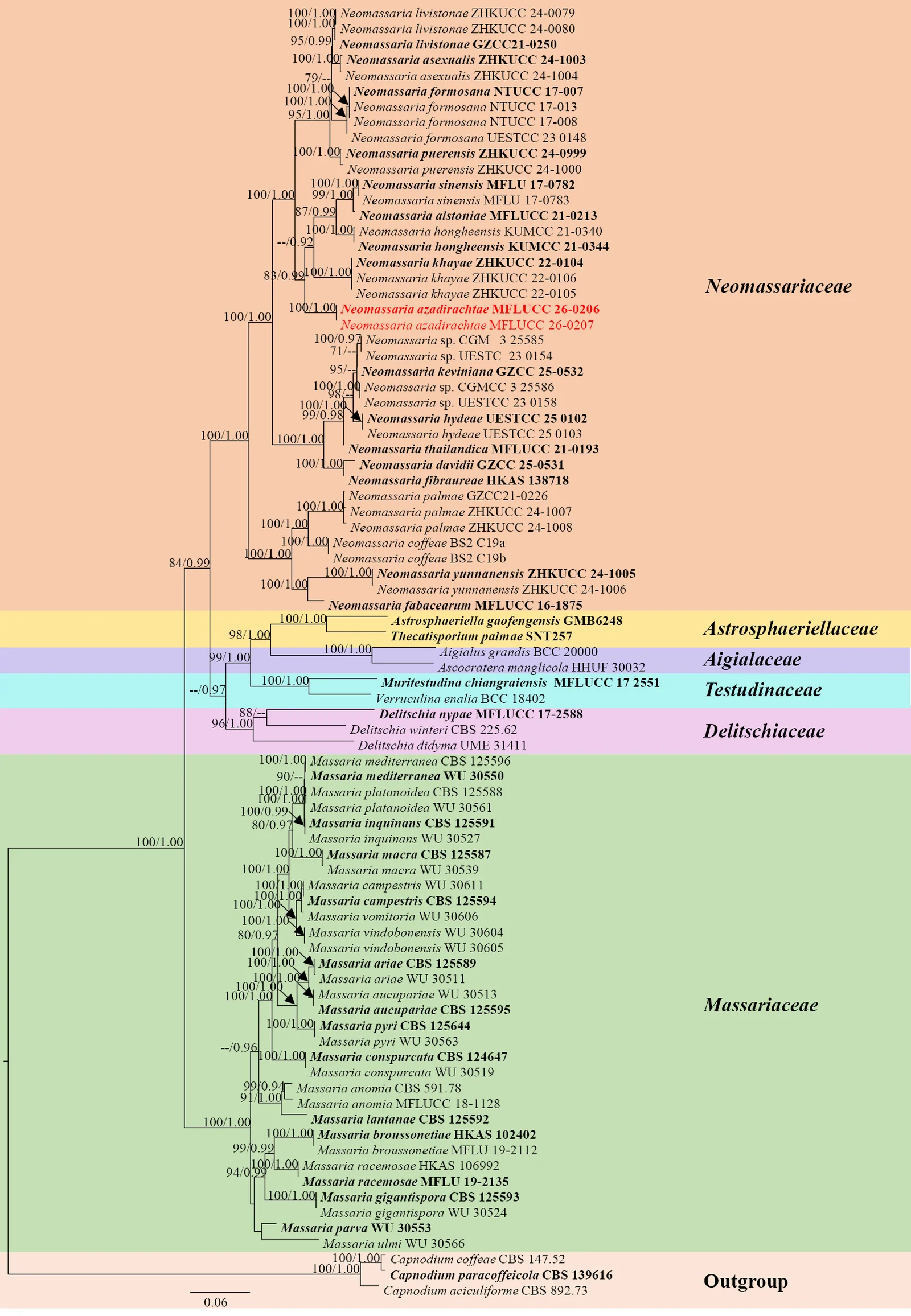
Phylogram generated from maximum likelihood analysis based on the LSU-SSU-*tef*1-α-*rpb*2 sequence matrix. The tree was rooted with *Capnodium coffeae* (CBS 147.52), *C. paracoffeicola* (CBS 139616), and *C. aciculiforme* (CBS 892.73). Bootstrap support values (ML ≥ 70%) and Bayesian posterior probabilities (≥ 0.80) are provided at the nodes. Type strains are in bold, and strains isolated in this study are in red.

In the phylogenetic analysis of *Neomassaria* strains, 83 taxa were included in the combined sequence dataset. *Capnodium coffeae* (CBS 147.52), *C. paracoffeicola* (CBS 139616), and *C. aciculiforme* (CBS 892.73) served as outgroup taxa. The aligned dataset comprised 5,256 characters, including gaps (LSU = 852, SSU = 1,965, *tef*1-α = 1,413, *rpb*2 = 1,026). Both ML and BI analyses produced similar tree topologies. The optimal RAxML tree yielded a final likelihood value of −34541.092 (Fig. 1). The matrix contained 2,061 distinct alignment patterns, with 37.49% undetermined or missing characters. Estimated base frequencies were as follows: A = 0.248, C = 0.243, G = 0.272, and T = 0.237. Substitution rates were AC = 1.46665, AG = 4.07932, AT = 1.0, CG = 1.46665, CT = 8.01627, and GT = 1.0. The gamma distribution shape parameter was *α* = 0.383. In the BI analysis, the burn-in percentage was 25%, and 3,750 trees were used to calculate PP. The average standard deviation of split frequencies in the BI analysis was 0.01 after 500,000 generations. Phylogenetic analyses indicated that *Neomassaria azadirachtae* (MFLUCC 26-0206 and MFLUCC 26-0207) formed a distinct sister clade to *Neomassaria sinensis* (MFLU 17-0782 and MFLU 17-0783), *Neomassaria alstoniae* (MFLUCC 21-0213), *Neomassaria hongheensis* (KUMCC 21-0344 and KUMCC 21-0340), and *Neomassaria khayae* (ZHKUCC 22-0104, ZHKUCC 22-0106, and ZHKUCC 22-0105), with 83% ML and 99 BPP support values (Fig. 1).

In the phylogenetic analysis of *Nigrograna* strains, 97 taxa were included in the combined sequence dataset. *Thyridaria aureobrunnea* (ZHKUCC 23-0075) and *Thyridariella mangrovei* (NFCCI 4213) served as outgroup taxa. The aligned dataset comprised 3,739 characters, including gaps (ITS = 537, LSU = 748, *rpb*2 = 1,054, SSU = 1,400). Both ML and BI analyses produced similar tree topologies. The optimal RAxML tree yielded a final likelihood value of −26037.769 (Fig. 3). The matrix contained 1,434 distinct alignment patterns, with 36.48% undetermined or missing characters. Estimated base frequencies were as follows: A = 0.253, C = 0.228, G = 0.272, and T = 0.247. Substitution rates were AC = 1.0, AG = 3.91747, AT = 1.0, CG = 1.0, CT = 7.51833, and GT = 1.0. The gamma distribution shape parameter was *α* = 0.328. In the BI analysis, the burn-in percentage was 25%, and 7,500 trees were used to calculate PP. The average standard deviation of split frequencies in the BI analysis was 0.006 after 1,000,000 generations. Phylogenetic analyses indicated that *Nigrograna azadirachtae* (MFLUCC 26-0225 and MFLUCC 26-0226) formed a distinct clade sister to *Nigrograna guttulata* (CGMCC 3.25689 and UESTCC 23.0295) and *Nigrograna peruviensis* (CCF 4485, B1a0908PD2CC348, C400610P15CC386, and HF12700) clade, with 86% ML and 0.86 BPP support values (Fig. 3).

**Figure 2.**
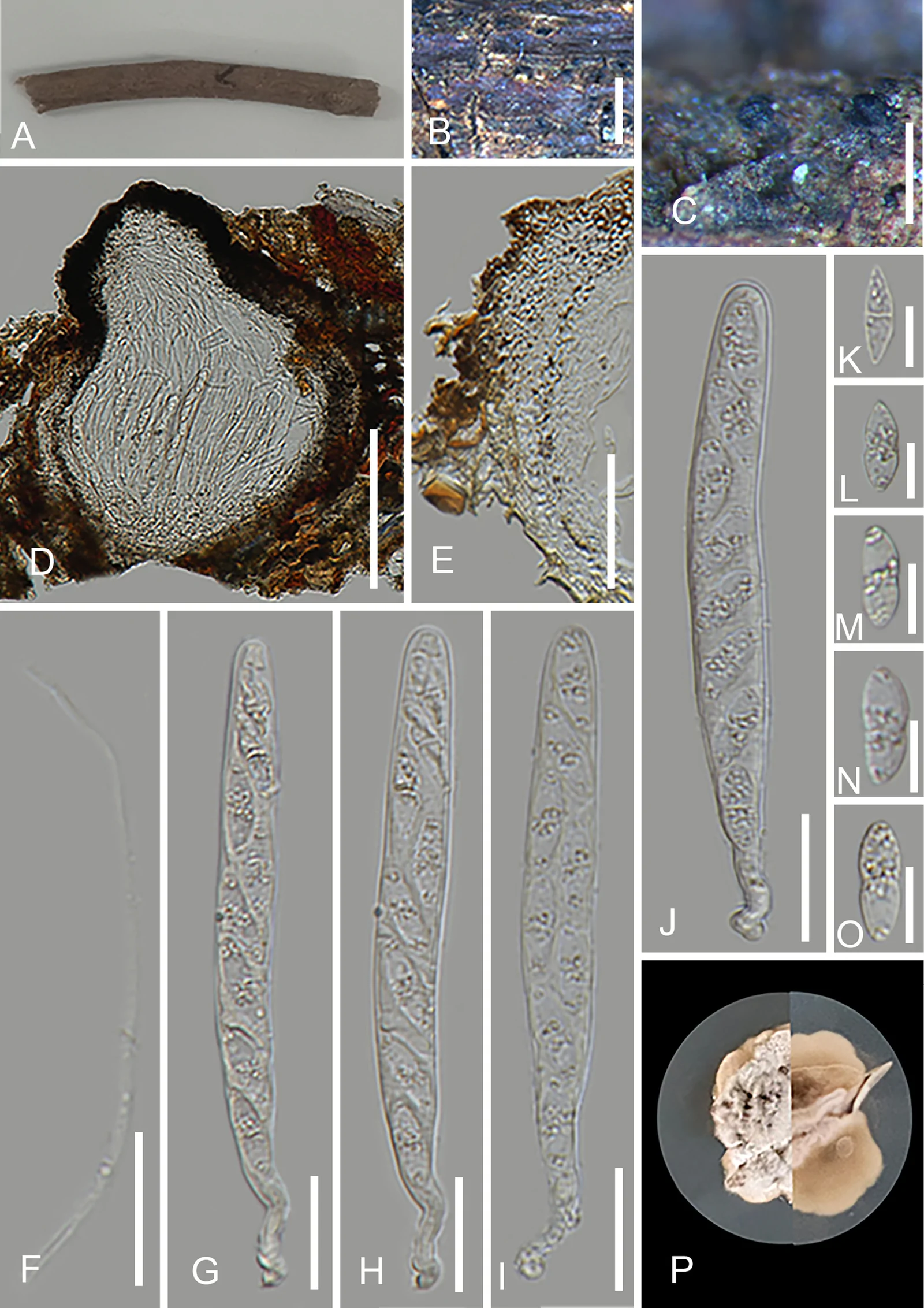
*Neomassaria azadirachtae* (MFLU 26-0213, holotype). **A**. Dead stem of *Azadirachta indica*; **B, C**. Appearance of ascomata on host substrate; **D**. Section of an ascoma; **E**. Peridium; **F**. Pseudoparaphyses; **G–J**. Asci; **K–O**. Ascospores; **P**. Fungal colony on PDA (upper: left; lower: right). Scale bars: 2.5 mm (**B**); 500 μm (**C**); 100 μm (**D**); 50 μm (**E**); 20 μm (**F–J**); 10 μm (**K–O**).

**Figure 3.**
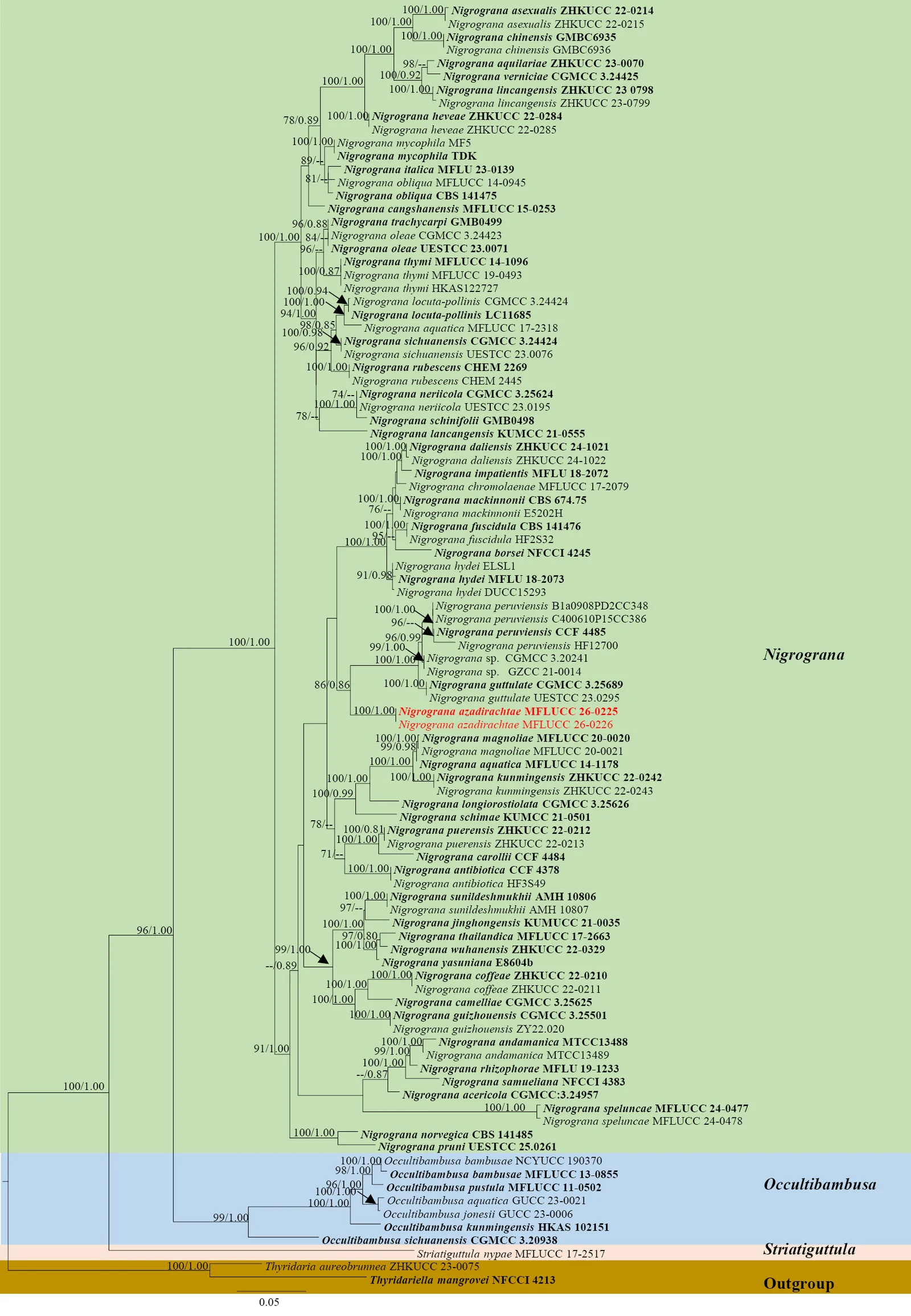
Phylogram generated from maximum likelihood analysis based on the ITS-LSU-*rpb*2-SSU sequence matrix. The tree was rooted with *Thyridaria aureobrunnea* (ZHKUCC 23-0075) and *T. mangrovei* (NFCCI 4213). Bootstrap support values (ML ≥ 70%) and Bayesian posterior probabilities (≥ 0.80) are provided at the nodes. Type strains are in bold, and strains isolated in this study are in red.

For the *Aquadictyospora yunnanensis* strains, 51 taxa were used for the phylogenetic analysis based on ITS, LSU, and *tef*1-α sequence data, with *P. igniaria* (CBS 298.66 and CBS 583.66) and *P. chiangraiensis* (MFLUCC 21-0164) as the outgroup taxa. After alignment, the combined sequence dataset comprised 2,184 characters, including gaps (ITS = 427, LSU = 857, *tef*1-α = 900). Both ML and BI analyses exhibited similar tree topologies. The best-scoring RAxML tree was obtained (Fig. 5), with a final likelihood value of −12341.407441. The matrix included 767 distinct alignment patterns, with 23.79% of characters undetermined or missing. Estimated base frequencies were as follows: A = 0.232494, C = 0.271672, G = 0.259890, and T = 0.235943. Substitution rates were AC = 1.409564, AG = 3.856492, AT = 1.946587, CG = 0.885173, CT = 8.567703, and GT = 1.0. The gamma distribution shape parameter was *α* = 0.235943. In the BI analysis, the burn-in percentage was 25%, and 75,000 trees were used to calculate PP. The average standard deviation of split frequencies in the BI analysis was 0.002 after 10,000,000 generations. According to the phylogenetic analyses, the *Aquadictyospora yunnanensis* strains (MFLUCC 26-0208 and MFLUCC 26-0209) formed a well-supported clade with the ex-type strain (ZHKUCC 22-0279) and another isolate (ZHKUCC 22-0280) of *Aquadictyospora yunnanensis* (Fig. 5).

**Figure 4.**
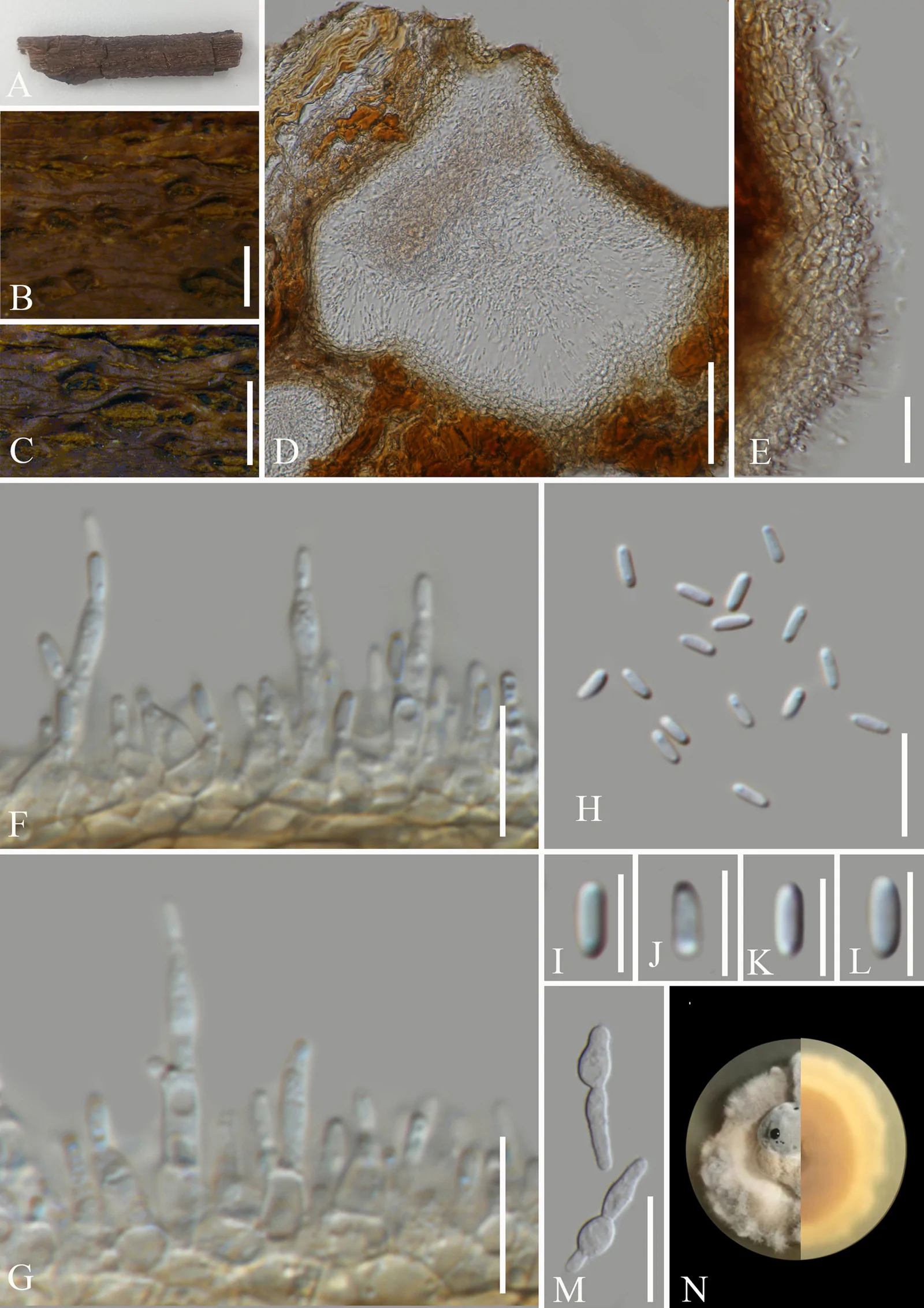
*Nigrograna azadirachtae* (MFLU 26-0294, holotype). **A**. Dead stem of *Azadirachta indica*; **B, C**. Appearance of ascomata on host substrate; **D**. Vertical section of a conidioma; **E**. Peridium; **F, G**. Conidiophores with phialides; **H–L**. Conidia; **M**. Germinating conidia; **N**. Fungal colony on PDA (upper: left; lower: right). Scale bars: 2.5 mm (**B, C**); 50 μm (**D**); 20 μm (**E, M**); 10 μm (**F–H**); 5 μm (**I–L**).

**Figure 5.**
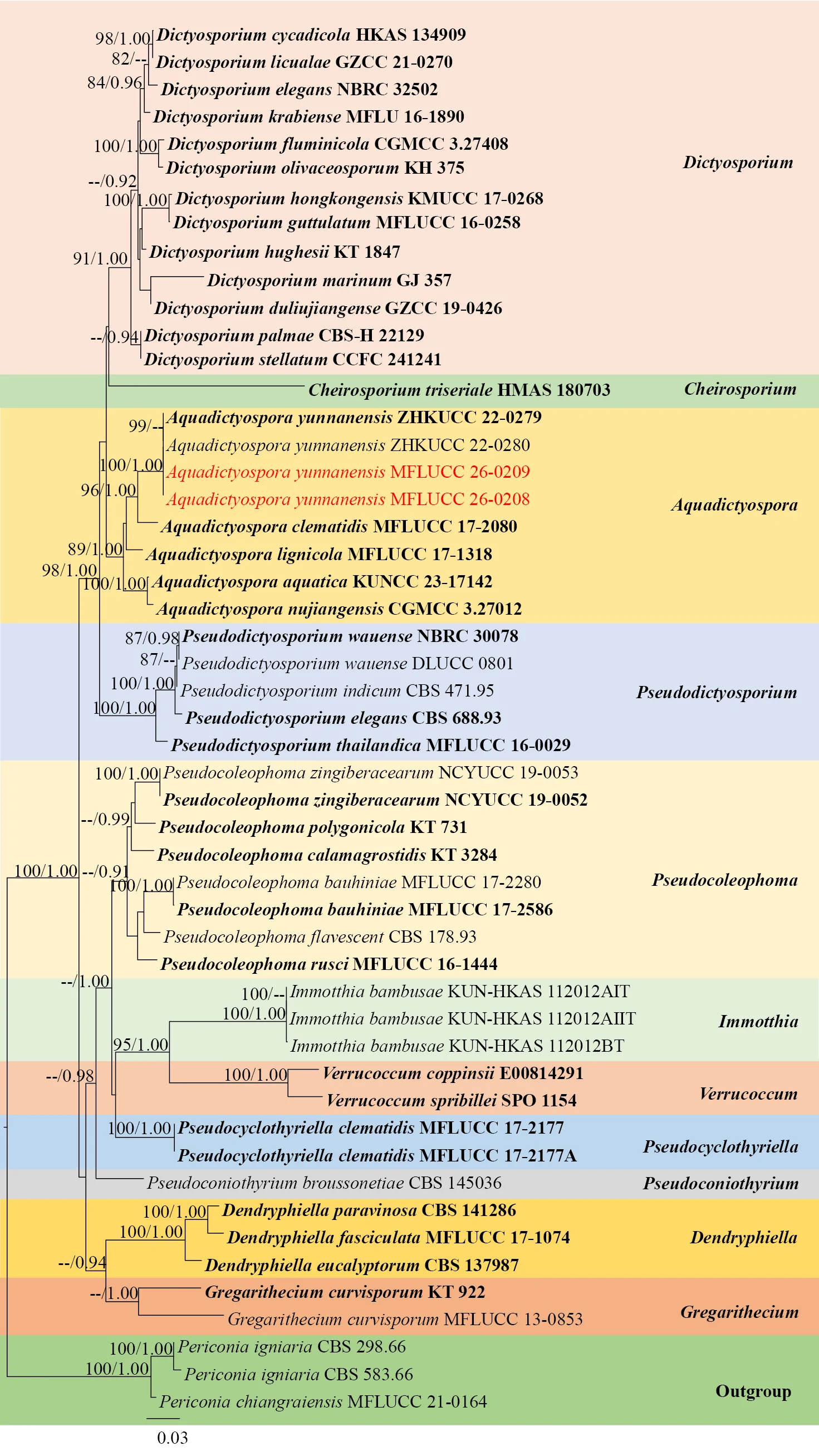
Phylogram generated from maximum likelihood analysis based on the ITS-LSU-*tef*1-α sequence matrix. The tree was rooted with *Periconia iginatia* (CBS 298.66 and CBS 583.66) and *P. chiangmaiensis* (MFLUCC 21-0164). Bootstrap support values (ML ≥ 75%) and Bayesian posterior probabilities (≥ 0.90) are provided at the nodes. Type strains are in bold, and strains isolated in this study are in red.

For the *Neoaquastroma bauhiniae* strain, 29 taxa were used for the phylogenetic analysis based on SSU, LSU, ITS, and *tef*1-α sequence data, with *Corynespora cassiicola* (CBS 100822 and CBS 161.60) as the outgroup taxon. The combined sequence dataset comprised 3,115 characters, including gaps (SSU = 969, LSU = 848, ITS = 385, *tef*1-α = 913). Both ML and BI analyses showed similar tree topologies. The best-scoring RAxML tree had a final likelihood value of −10174.212615. The matrix included 603 distinct alignment patterns, with 12.91% of characters undetermined or missing. Estimated base frequencies were as follows: A = 0.234705, C = 0.257438, G = 0.274991, and T = 0.232866. Substitution rates were AC = 1.105892, AG = 2.147829, AT = 1.216706, CG = 1.397512, CT = 7.660876, and GT = 1.0. The gamma distribution shape parameter was *α* = 0.116870. In the BI analysis, the burn-in was set to 25%, and 62,400 post-burn-in trees were retained for calculating PP. The analysis terminated automatically after 8,320,000 generations when the average standard deviation of split frequencies reached 0.001. In the phylogenetic tree, the *Neoaquastroma bauhiniae* strain (MFLUCC 26-0210) clustered with the ex-type strain (MFLU 17-0002) and another isolate (MFLU 17-0559) of *Neoaquastroma bauhiniae* (Fig. 7).

**Figure 6.**
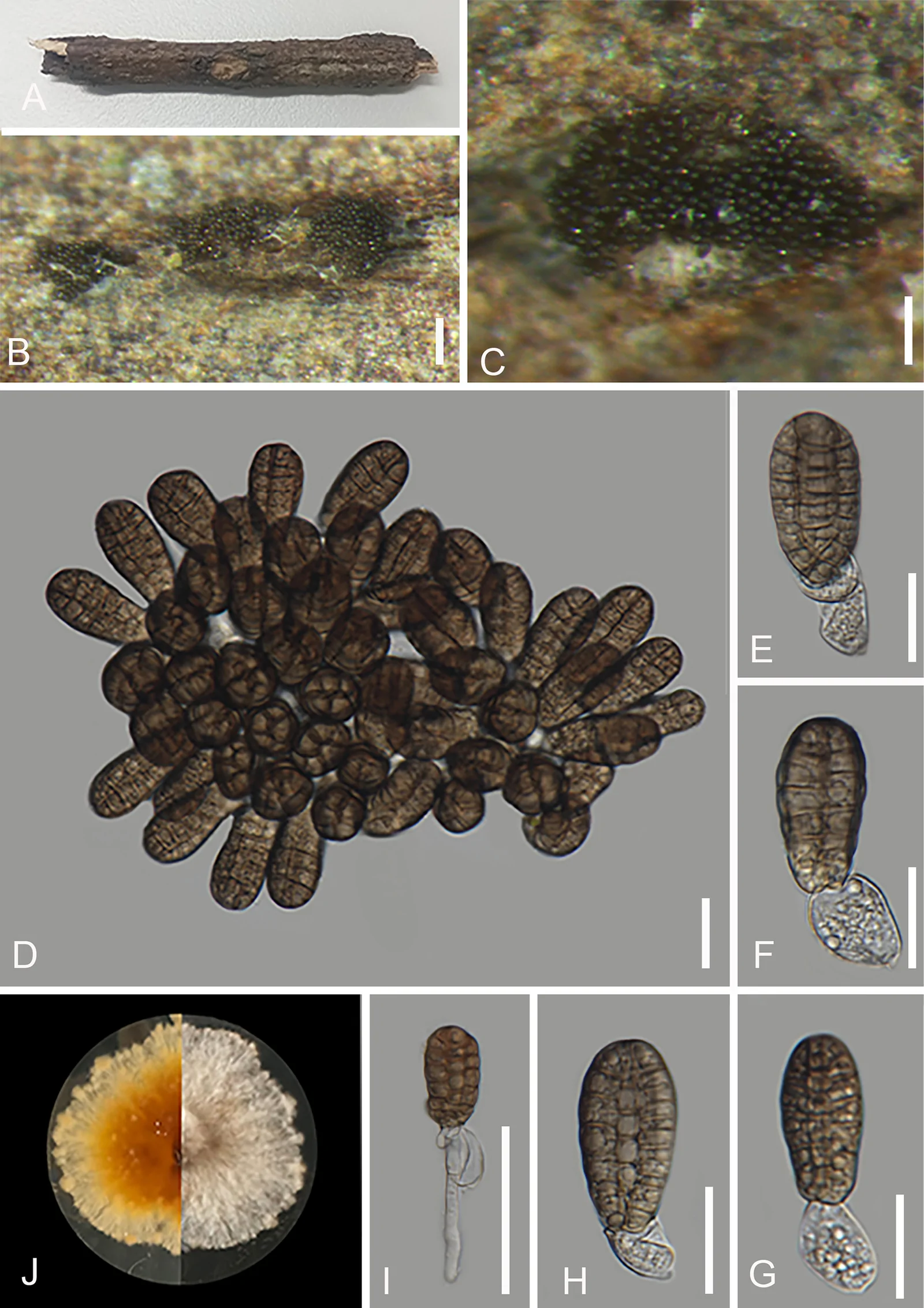
*Aquadictyospora yunnanensis* (MFLU 26-0214). **A**. A dead stem of *Azadirachta indica*; **B, C**. Sporodochia on natural substrate; **D**. Squash mount of conidia; **E–H**. Mature conidia attached to basal cells; **I**. A germinating conidium; **J**. Fungal colony on PDA (upper: left; lower: right). Scale bars: 100 μm (**B**); 75 μm (**C**); 20 μm (**D–H**); 50 μm (**I**).

**Figure 7.**
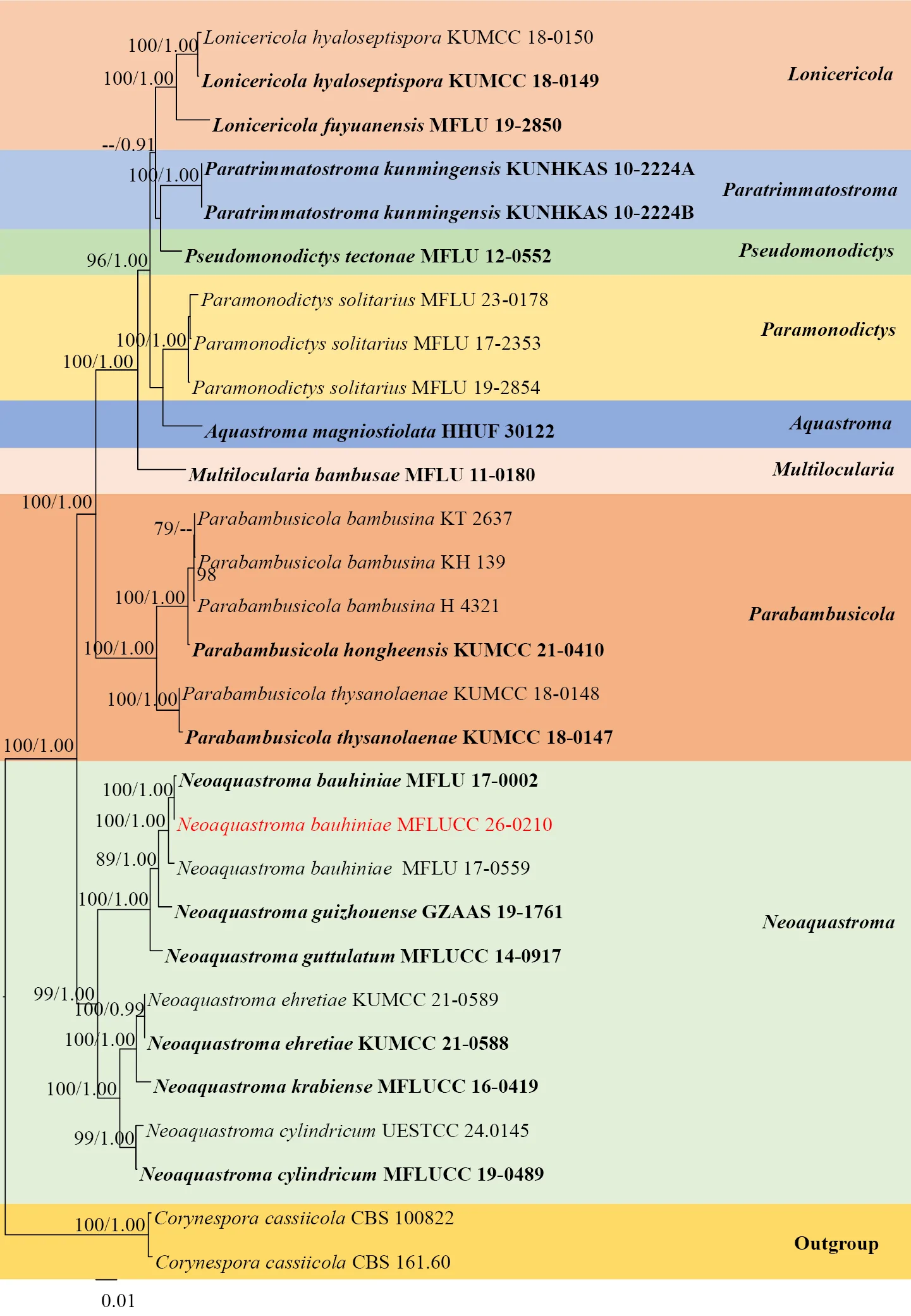
Phylogram generated from maximum likelihood analysis based on the SSU-LSU-ITS-*tef*1-α sequence matrix. The tree was rooted with *Corynespora cassiicola* (CBS 100822 and CBS 161.60). Bootstrap support values (ML ≥ 80%) and Bayesian posterior probabilities (≥ 0.90) are provided at the nodes. Type strains are in bold, and the strain isolated in this study is in red.

In the phylogenetic analysis of the *Pararoussoella lincangensis* strain, 54 taxa were included in the combined sequence dataset. *Torula herbarum* (CBS 111855) and *Torula hollandica* (CBS 22.069) were used as the outgroup taxa. The combined dataset comprised 3,917 characters, including gaps (LSU = 908, ITS = 982, *tef*1-α = 939, *rpb*2 = 1,088). Both ML and BI analyses showed similar tree topologies. The best-scoring RAxML tree (Fig. 9) had a final likelihood value of −26268.399417. The matrix included 1,710 distinct alignment patterns, with 37.38% of characters undetermined or missing. Estimated base frequencies were as follows: A = 0.241359, C = 0.265129, G = 0.270972, and T = 0.222541. Substitution rates were AC = 1.468751, AG = 3.930911, AT = 1.650361, CG = 1.116481, CT = 8.455054, and GT = 1.0. The gamma distribution shape parameter was *α* = 0.218330. In the BI analysis, the burn-in percentage was 25%, and 75,000 trees were used to calculate PP. The average standard deviation of split frequencies in the BI analysis was 0.005 after 10,000,000 generations. In the phylogenetic tree, the strain MFLUCC 26-0211 clustered with the ex-type strain (KUMCC 21-0619), other strains (KUMCC 21-0620, MFLUCC 24-0532, MFLUCC 24-088, and MFLUCC 24-089) of *Pararoussoella lincangensis* as well as two strains of *Pararoussoella* sp. (UESTCC:23.0423 and UESTCC:23.0422), and formed a well-supported clade (100% ML and 1.00 BPP) (Fig. 9).

**Figure 8.**
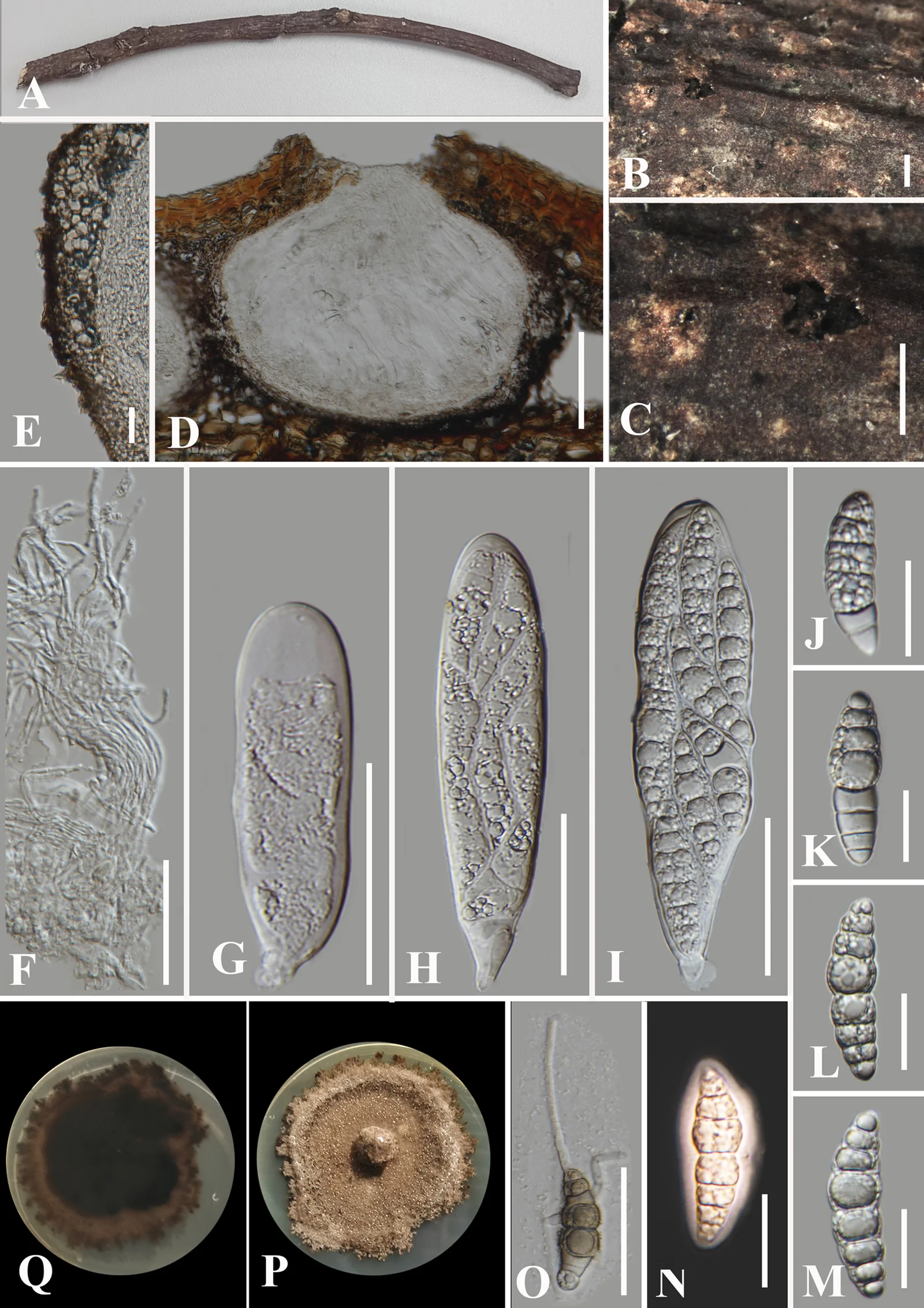
*Neoaquastroma bauhiniae* (MFLU 26-0215). **A**. Dead stems of *Azadirachta indica*; **B, C**. Appearance of ascomata on host substrate; **D**. Section of an ascoma; **E**. Peridium; **F**. Pseudoparaphyses; **G–I**. Asci; **J–N**. Ascospores; **O**. A germinating ascospore; **P, Q**. Fungal colony on PDA (upper: **P**; lower: **Q**). Scale bars: 1 mm (**B, C**); 100 μm (**D**); 20 μm (**E, J–N**); 50 μm (**F–I, O**).

**Figure 9.**
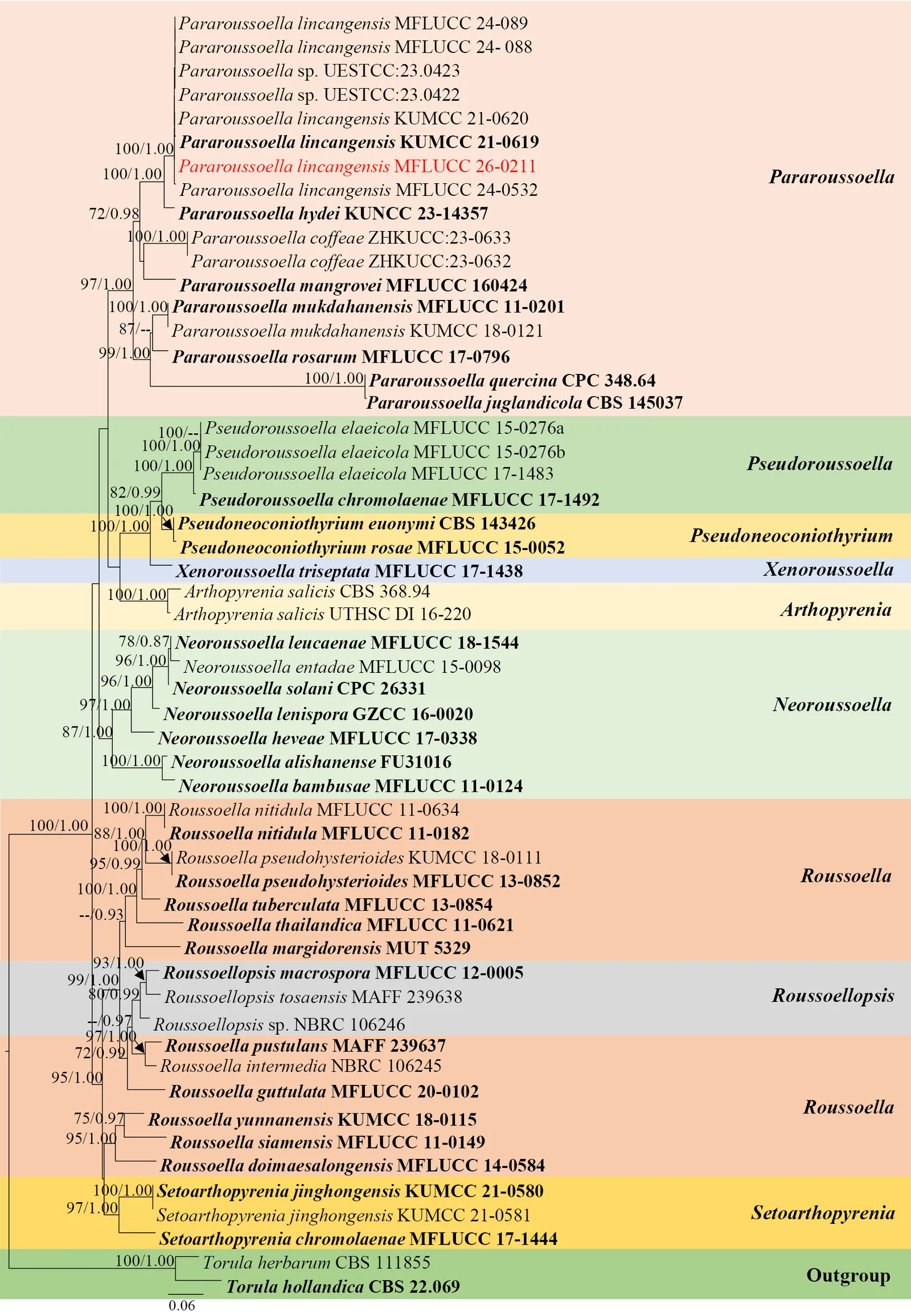
Phylogram generated from maximum likelihood analysis based on the LSU-ITS-*tef*1-α-*rpb*2 sequence matrix. The tree was rooted with *Torula herbarum* (CBS 111855) and *Torula hollandica* (CBS 22.069). Bootstrap support values (ML ≥ 70%) and Bayesian posterior probabilities (≥ 0.80) are provided at the nodes. Type strains are in bold, and the strain isolated in this study is in red.

### Taxonomy

#### Neomassaria

Taxon classificationFungiPleosporalesNeomassariaceae

Mapook, Camporesi & K.D. Hyde

A21FEB44-54E7-5048-856C-62A28542105D

##### Notes.

Hyde et al. (2016) originally introduced *Neomassaria* under *Massariaceae*, with *N. fabacearum* as the type species, based on an isolate from a dead branch of *Hippocrepis emerus* in Italy. Later, based on phylogenetic analyses of LSU, *rpb*2, SSU, and *tef*1-α sequence data, Ariyawansa et al. (2018) introduced *N. formosana* and recognised *Neomassaria* as a monophyletic, well-supported clade within *Pleosporales*, distinct from other families in the order. Based on their results, Ariyawansa et al. (2018) introduced *Neomassariaceae* to accommodate *Neomassaria* as the type genus of this family. Currently, Index Fungorum (2026) lists 17 species, and these species have been reported as saprobes on various hosts in Thailand, China, and Italy (Dong et al. 2025; Lu et al. 2025; Sun et al. 2025; Xiao et al. 2025). In this study, a new species, *Neomassaria azadirachtae*, is introduced from neem (*Azadirachta indica*) in Thailand.

#### Neomassaria azadirachtae

Taxon classificationFungiPleosporalesNeomassariaceae

Madagammana, Bhunjun & K.D. Hyde sp. nov.

8B2B3163-8DA0-5352-A814-FA74984B7E7E

Index Fungorum: IF905329

Facesoffungi Number: FoF20074

Fig. 2

##### Etymology.

The specific epithet “*azadirachtae*” refers to the host genus *Azadirachta* of the holotype.

##### Type.

Thailand • Chiang Rai Province, Nang Lae (20.053697°N, 99.894733°E), on dead stems of *Azadirachta indica* (*Meliaceae*), 16 September 2024, Ashani D. Madagammana, AD 0704, (MFLU 26-0213, holotype), ex-type living culture = MFLUCC 26-0206.

##### Description.

***Saprobic*** on dead stems of *Azadirachta indica* A. Juss. **Sexual morph: *Ascomata*** 220–250 × 200–220 µm (x̄ = 230 × 210 µm, *n* = 10), semi-immersed, scattered to gregarious, pyriform to subglobose, unilocular, coriaceous, dark brown to black, ostiolate. ***Ostiole*** central, erumpent, forming black, slightly raised areas on the host surface. ***Peridium*** 15–45–µm wide, composed of several layers of dark brown thick-walled cells of *textura angularis*, becoming hyaline to pale brown and thin-walled towards the inner layers. Apical region black, pigmented, thick-walled with a multilayered clypeus. Darkened outer layers almost disappear at the base, where fused with the host. ***Hamathecium*** 1–2–µm wide, consists of hyaline, branched, filamentous pseudoparaphyses, embedded in a gelatinous matrix. ***Asci*** 90–120 × 9–12 µm (x̄ = 104 × 10.7 µm, *n* = 20), 8-spored, bitunicate, fissitunicate, overlapping 1–2-seriate, cylindrical to cylindrical-clavate, straight, apically rounded, slightly long pedicellate. ***Ascospores*** 15–18 × 4–7 µm (x̄ = 16.6 × 5.1 µm, *n* = 20; *L/W* = 3.3), ellipsoid to ovoid, hyaline, 1-septate in the middle, constricted at the septum, smooth-walled, broadens at the upper region, subacute, with granules, without a mucilaginous sheath. **Asexual morph**: Undetermined.

##### Culture characteristics.

Colonies on PDA reaching 5 mm diam. after 2 weeks at 25 °C, dense, irregular, surface cottony to floccose, uneven and wrinkled, with irregular folds; raised areas white to greyish-white, with intervening depressed areas pale beige to light brown; margin entire to slightly undulate; reverse inner zone darker brown, becoming pale brown to tan towards the outer zone.

##### Material examined.

Thailand • Chiang Rai Province, Nang Lae, dead stems of *Azadirachta indica* A. Juss. (*Meliaceae*), 16 September 2024, Ashani D. Madagammana, AD 0700 (MFLU 26-0213, holotype); ex-type living culture, (AD 0700A) MFLUCC 26-0206; living culture, (AD 0700B) MFLUCC 26-0207.

##### GenBank accession numbers.

MFLUCC 26-0206: SSU: PZ465540, LSU: PZ456340, *rpb*2: PZ463027, *tef*1-α: PZ495682; MFLUCC 26-0207: SSU: PZ465541, LSU: QB046478, *tef*1-α: PZ495683.

##### Notes.

Based on the multi-locus phylogenetic analysis, our strains MFLUCC 26-0206 and MFLUCC 26-0207 formed a distinct sister clade to *Neomassaria sinensis* (MFLU 17-0782 and MFLU 17-0783), *Neomassaria alstoniae* (MFLUCC 21-0213), *Neomassaria hongheensis* (KUMCC 21-0344 and KUMCC 21-0340), and *Neomassaria khayae* (ZHKUCC 22-0104, ZHKUCC 22-0106, and ZHKUCC 22-0105), with 83% ML and 99 BPP support (Fig. 1). Morphologically, our species resembles *N. sinensis*, *N. alstoniae*, *N. hongheensis*, and *N. khayae*, with semi-immersed, dark brown to black, ostiolate ascomata, filamentous pseudoparaphyses, bitunicate asci, and hyaline ascospores (de Silva et al. 2022; Yang et al. 2022, 2023; Dong et al. 2025). The morphological and nucleotide differences between our strains and closely related species are listed in Table 2. Based on morphological differences and multi-locus phylogenetic analyses, our strains represent a distinct clade and are proposed here as a new species.

**Table 2.** Morphological and nucleotide differences between the new species and its phylogenetically close relatives.

**Species**	**Morphological differences from *Neomassaria azadirachtae***	**Reference(s)**	**LSU**	**SSU**	***tef*1-α**	***rpb*2**
*Neomassaria sinensis*	Smaller ascomata (100–150 × 120–200 µm vs. 220–250 × 200–220 µm), shorter asci (40–75 × 8–10 µm vs. 90–120 × 9–12 µm), and predominantly curved ascospores vs. straight	(Dong et al. 2025)	2.35–2.72%	6.09–8.83%	7.70%	–
*N. alstoniae*	Wider asci (12–18 µm vs. 9–12 µm) and larger ascospores (20–24 × 7–10 µm vs. 15–18 × 4–7 µm)	(de Silva et al. 2022)	2.35–2.72%	5.51–8.68%	–	–
*N. hongheensis*	Dome-shaped ascomata vs. pyriform to subglobose; lower ascospore L/W ratio (2.5 vs. 3.3)	(Yang et al. 2022)	2.09–2.56%	0.80–1.23%	5.99–6.02%	12.73–13.03%
*N. khayae*	*Textura prismatica* outer peridial layer and *textura subcircular* inner layer vs. *textura angularis*; 1–2-septate ascospores vs. 1-septate; longer ascospores (25–35 × 2–8 µm vs. 15–18 × 4–7 µm)	(Yang et al. 2023)	2.08–2.41%	0.30–0.35%	5.43%	14.23%

N.B.: Pairwise nucleotide differences (%) were calculated between *N. azadirachtae* (MFLUCC 26-0206 and MFLUCC 26-0207) and *N. sinensis* (MFLU 17-0782 and MFLU 17-0783), *N. alstoniae* (MFLUCC 21-0213), *N. hongheensis* (KUMCC 21-0344 and KUMCC 21-0340), and *N. khayae* (ZHKUCC 22-0104, ZHKUCC 22-0106, and ZHKUCC 22-0105); the *rpb*2 comparison with *N. khayae* and *N. hongheensis* was based on MFLUCC 26-0206 only.

#### Nigrograna

Taxon classificationFungiPleosporalesNigrogranaceae

Gruyter, Verkley & Crous

CEC80A58-3B9D-5DBD-BA9A-2D0CDF123CEA

##### Notes.

De Gruyter et al. (2013) introduced *Nigrograna* as a monophyletic genus, with *N. mackinnonii* as the type species. Later, Ahmed et al. (2014) synonymised *Nigrograna* with *Biatriospora* and transferred *N. mackinnonii* to *Biatriospora*, based on molecular data similarities with the type species. Subsequently, Jaklitsch and Voglmayr (2016) re-established *Nigrograna*, added new species to the genus, and established *Nigrogranaceae* to accommodate it. Kolařík (2018) subsequently reassigned several endophytic species previously placed in *Biatriospora* to *Nigrograna*. Since then, numerous additional species have been added to this genus, and currently, *Nigrograna* comprises 51 accepted species in Index Fungorum (2026). Species of this genus are endophytes, saprobes, or human pathogens in various habitats, such as dead wood, hive-stored pollen, stromatic fungi, human eumycetomas, healthy trees, marine environments, and soil, in various countries, including Austria, China, Italy, Norway, Thailand, and Venezuela (De Gruyter et al. 2013; Jaklitsch and Voglmayr 2016; Kolařík et al. 2017; Zhao et al. 2018; Phukhamsakda et al. 2020; Boonmee et al. 2021; Mack et al. 2024; Yasanthika et al. 2025).

#### Nigrograna azadirachtae

Taxon classificationFungiPleosporalesNigrogranaceae

Madagammana, Bhunjun & K.D. Hyde sp. nov.

207102FB-295B-52AB-93FD-0469DADADF6A

Index Fungorum: IF905192

Facesoffungi Number: FoF20166

Fig. 4

##### Etymology.

The specific epithet “*azadirachtae*” refers to the host genus “*Azadirachta*”.

##### Type.

Thailand • Chiang Rai Province, Nang Lae (20.053697°N, 99.894733°E), on dead stems of *Azadirachta indica* A. Juss. (*Meliaceae*), 14 October 2024, Ashani D. Madagammana, AD 0704, (MFLU 26-0294, holotype), ex-type living culture = MFLUCC 26-0225.

##### Description.

***Saprobic*** on dead stems of *Azadirachta indica*. **Sexual morph**: Undetermined. **Asexual morph**: Coelomycetous. ***Pycnidia*** 185–485 × 170–380 µm (x̄ = 260 × 225 µm, *n* = 05), solitary, immersed, brown to dark brown, unilocular, locules variable in shape, globose to subglobose, compressed to irregularly elongated. ***Peridium*** 19–29 µm wide (x̄ = 24 μm, *n* = 15), 4–8-layered, composed of *textura angularis* cells, outer layer cells pale brown to brown, thick-walled, inner layers hyaline. ***Conidiophores*** 10–30 × 1.5–3 μm (x̄ = 18 × 2.2 μm, *n* = 15), filiform, septate, hyaline, simple to sparsely branched, arising in a palisade from the inner wall of the conidioma, with short peg-like lateral conidiogenous extensions occasionally present on one or both sides and terminating in a solitary phialide at the conidiophore apex; both the lateral conidiogenous extensions and the terminal phialide produce a solitary conidium. ***Phialides*** 5–9 × 1.5–3 µm (x̄ = 6.8 × 2.1 µm, *n* = 15), variable in shape, discrete, ampulliform-lageniform-subcylindrical. ***Conidia*** 3.5–4.3 × 1.0–1.70 μm (x̄ = 3.9 × 1.4 μm, *n* = 50; *L/W* = 2.8), hyaline, aseptate, smooth-walled, ellipsoidal to short-cylindrical, with broadly rounded ends, 1-celled, containing 1–2 guttules.

##### Culture characteristics.

Colonies on PDA reaching 25 mm diam. after 2 months at 20–25 °C, dense, circular, surface cottony to floccose, centrally umbonate with irregularly elevated folds; raised folds grey to dark grey, separated by intervening depressed areas that are white to pale grey; margin entire to slightly undulate; guttation droplets produced at the centre; reverse inner zone cinnamon brown; outer zone cream to pale yellow.

##### Material examined.

Thailand • Chiang Rai Province, Nang Lae, dead stems of *Azadirachta indica* A. Juss. (*Meliaceae*), 14 October 2024, Ashani D. Madagammana, AD 0704 (MFLU 26-0294, holotype); ex-type living culture (AD 0704 A), MFLUCC 26-0225; living culture (AD 0704 B), MFLUCC 26-0226.

##### GenBank accession numbers.

MFLUCC 26-0225: ITS: PZ631856, LSU: PZ631860, *rpb*2: PZ671299, SSU: PZ631858; MFLUCC 26-0226: ITS: PZ631857, LSU: PZ631861, *rpb*2: PZ671300, SSU: PZ631859.

##### Notes.

Based on the multi-locus phylogenetic analysis, our strains MFLUCC 26-0225 and MFLUCC 26-0226 formed a distinct clade with 86% ML and 0.86 BPP support (Fig. 3), sister to the *Nigrograna guttulata* (CGMCC 3.25689 and UESTCC 23.0295) and *N. peruviensis* (CCF 4485, B1a0908PD2CC348, C400610P15CC386, and HF12700) clade. As *Nigrograna guttulata* was described only from its sexual morph and our species was observed only in its asexual morph, their morphologies could not be compared. Kolařík et al. (2017) first reported *Nigrograna peruviensis* as *Biatriospora peruviensis*, and Kolařík (2018) later synonymised it under *Nigrograna*. However, due to the lack of detailed morphological descriptions, this species could not be compared with our species. Morphologically, the new species shares common coelomycetous asexual morph characters with *Nigrograna* species (e.g. *N. asexualis*, *N. magnoliae*, and *N. fuscidula*), including hyaline, septate conidiophores arranged in a palisade with diagnostic lateral pegs and terminal phialides that produce smooth, 1-celled, guttulate conidia, but is distinguished by its independent phylogenetic placement based on multi-locus analyses (Jaklitsch and Voglmayr 2016; Wanasinghe et al. 2020; Lu et al. 2022). In addition, *Nigrograna azadirachtae* (MFLUCC 26-0225 and MFLUCC 26-0226) differs from *N. guttulata* (CGMCC 3.25689 and UESTCC 23.0295) by 7.7–7.8% in ITS, 3.24% in LSU, and 15.79–15.82% in *rpb*2. It differs from *Nigrograna peruviensis* (CCF 4485, B1a0908PD2CC348, C400610P15CC386, and HF12700) by 7.9–11.5% in ITS. For LSU and *rpb*2, sequence comparisons were possible only with strain CCF 4485, which showed 2.66% nucleotide difference in LSU and 16.13–16.31% nucleotide differences in *rpb*2. However, no nucleotide differences were detected in the SSU region between our strains and either *Nigrograna guttulata* or *N. peruviensis*, suggesting that SSU provides limited variation among these closely related taxa. Based on the observed morphological characteristics and multi-gene phylogenetic analyses, our strains represent a distinct clade and are introduced here as a new species, *Nigrograna azadirachtae*.

#### Aquadictyospora

Taxon classificationFungiPleosporalesDictyosporiaceae

Z.L. Luo, K.D. Hyde & Hong Y. Su

AC468FDB-A47F-5B37-BB64-8CDADBF93FCC

##### Notes.

Li et al. (2017) introduced *Aquadictyospora* based on morpho-molecular data, with *A. lignicola* as the type species. This genus is currently listed under *Dictyosporiaceae*, *Pleosporales*, and *Dothideomycetes* (Boonmee et al. 2016; Hongsanan et al. 2020; Hyde et al. 2024b). Members of this genus are characterised by having sporodochia, superficial, circular or subglobose conidiomata, and micronematous conidiophores with monoblastic conidiogenous cells (Phukhamsakda et al. 2020; Liu et al. 2024; Zhang et al. 2024). Their conidia are uniformly medium brown, dictyosporous, and attached to a subglobose, hyaline cell at the basal end (Phukhamsakda et al. 2020; Liu et al. 2024; Zhang et al. 2024). To date, no sexual morph has been reported for any species of this genus. Currently, Species Fungorum (2026) lists five species, all reported from submerged decayed wood in freshwater habitats and from dead stems (*Coffea arabica* and *Clematis sikkimensis*); four were originally reported from China and one from Thailand (Phukhamsakda et al. 2020; Han et al. 2024; Zhang et al. 2024).

#### Aquadictyospora yunnanensis

Taxon classificationFungiPleosporalesDictyosporiaceae

L. Lu, M.Y. Han & Tibpromma, in Han, Lu, Karunarathna, Zheng, Elgorban & Tibpromma, Phytotaxa 674: 3 (2024)

2CC67D10-4F84-5630-B42E-D38BCB25F477

Index Fungorum: IF902532

Facesoffungi Number: FoF16620

Fig. 6

##### Type.

China • Yunnan Province, Jinghong City, a decaying branch of *Coffea arabica*, (22°31'18"N, 101°2'44"E, 856.89 m), 15 September 2021, LiLu, JHPW7 (MHZU 22-0148, holotype), ex-type living culture (ZHKUCC 22-0279).

##### Description.

***Saprobic*** on dead stems of *Azadirachta indica*. **Sexual morph**: Undetermined. **Asexual morph**: Hyphomycetous. ***Colonies*** on natural substrate sporodochial, superficial, velvety, dark brown to black, subglobose, scattered in small groups, crowded and confluent when older, with base attached to the surface of the host plant. ***Mycelium*** immersed, septate, branched and hyaline hyphae. ***Conidiophores*** micronematous, reduced to conidiogenous cell, hyaline to pale brown. ***Conidiogenous cells*** holoblastic, solitary, and determinate. ***Conidia*** 24–36 × 14–20 μm (x̄ = 31.9 × 16.6 μm, *n* = 20), dictyosporous, oval to obovoid, compactly adpressed, 2–4 apically rows of cells, guttulate, with basal connecting cell 14–22 × 12–15 μm (x̄ = 17 × 13 μm, *n* = 20), hyaline and subglobose or ovoid, not complanate, secession involves splitting of the basal, without appendages or mucilaginous sheath.

##### Culture characteristics.

Colonies on PDA reaching 50 mm diam. after 2 months at 20–25 °C, dense, circular, surface floccose to woolly, flat; white to cream, with yellowish to brown pigmentation centrally; margin lobate, irregular, and slightly undulate; reverse yellow to golden yellow, becoming orange-brown towards the centre.

##### Material examined.

Thailand • Chiang Rai Province, Nang Lae, dead stems of *Azadirachta indica* A. Juss. (*Meliaceae*), 19 August 2024, Ashani D. Madagammana, AD 0701 (MFLU 26-0214); living cultures, (AD 0701A) MFLUCC 26-0208, (AD 0701B) MFLUCC 26-0209.

##### GenBank accession numbers.

MFLUCC 26-0208: ITS: PZ445649, LSU: PZ456341, *tef*1-α: PZ495685; MFLUCC 26-0209: ITS: PZ445650, LSU: PZ456342, *tef*1-α: PZ495684.

##### Notes.

Based on the phylogenetic analysis, our strains MFLUCC 26-0208 and MFLUCC 26-0209 grouped with the ex-type strain (ZHKUCC 22-0279) and another isolate (ZHKUCC 22-0280) of *A. yunnanensis* with 100% ML and 1.00 BPP support (Fig. 5). Our isolates are morphologically similar to the holotype of *A. yunnanensis* in having micronematous conidiophores that are reduced to conidiogenous cells, holoblastic conidiogenous cells, and dictyosporous, oval to obovoid conidia with a hyaline basal cell and lacking appendages or a mucilaginous sheath (Han et al. 2024). However, the basal connecting cell is larger in our isolates than in the holotype (14–22 × 12–15 μm; x̄ = 17 × 13 μm vs. 10–15 × 9–11 μm; x̄ = 12 × 10 μm) (Han et al. 2024). The type collection of this species was collected from a decaying branch of *Coffea arabica* in Yunnan Province, China (Han et al. 2024). The strains (MFLUCC 26-0208 and MFLUCC 26-0209) were identified as *A. yunnanensis* based on morphological and phylogenetic analyses. Previously, this species was reported only on *Coffea arabica* in Yunnan Province. In this study, a new host record from *Azadirachta indica* and a new geographical record from Thailand are provided. Because no sexual morph has been reported for this species, increased taxon sampling from different countries, including the type locality, is recommended to better understand *A. yunnanensis*.

#### Neoaquastroma

Taxon classificationFungiPleosporalesParabambusicolaceae

Wanas., E.B.G. Jones & K.D. Hyde

1D119465-7F82-5D49-B27B-BE87F66778CE

##### Notes.

Based on morphology and molecular data, Wanasinghe et al. (2017) introduced *Neoaquastroma* in *Parabambusicolaceae*, with *N. guttulatum* as the type species, collected on dead herbaceous stems in Thailand. Later, five more species were introduced to this genus, namely *N. bauhiniae*, *N. krabiense*, *N. cylindricum*, *N. guizhouense*, and *N. ehretiae* (Phukhamsakda et al. 2018; Samarakoon et al. 2019; Zhang et al. 2023; Ren et al. 2024). *Neoaquastroma* is characterised by immersed, brown to dark brown ascomata; bitunicate, clavate asci; and hyaline, multi-septate, ellipsoidal to subfusiform, guttulate ascospores with a distinct mucilaginous sheath (Wanasinghe et al. 2017). Initially, *Neoaquastroma* (*N. guttulatum*) was reported only as a sexual morph, but Phukhamsakda et al. (2018) later described both sexual and asexual morphs for *N. bauhiniae* and *N. krabiense*, with the asexual morphs characterised by conidiomata producing aseptate, hyaline conidia. *Neoaquastroma* shows similarities with *Parabambusicola* and *Multiseptospora*. However, *Neoaquastroma* forms a well-supported clade in multi-gene phylogenetic analyses and differs from these genera in ascospore morphology, particularly in ascospore size and number of septa (Phukhamsakda et al. 2018). Currently, six species are listed in Index Fungorum (2026), and all have been reported as saprobes, of which three species were originally reported from China and three from Thailand (Wanasinghe et al. 2017; Phukhamsakda et al. 2018; Samarakoon et al. 2019; Zhang et al. 2023; Ren et al. 2024).

#### Neoaquastroma bauhiniae

Taxon classificationFungiPleosporalesParabambusicolaceae

Phukhams. & K.D. Hyde, in Phukhamsakda, Bhat, Hongsanan, Xu, Stadler & Hyde, MycoKeys 34: 54 (2018)

89AD74DD-CE0C-531D-9216-FFB73734707B

Index Fungorum: IF824673

Facesoffungi Number: FoF04371

Fig. 8

##### Type.

Thailand • Phrae Province, Song District, on dead twigs of *Bauhinia variegata* L. (*Fabaceae*), 25 July 2015, C. Phukhamsakda, holotype MFLU 17-0002 (S1-11); ex-type living culture MFLUCC 16-0398 (= ICMP 21572).

##### Description.

***Saprobic*** on dead stems of *Azadirachta indica*. **Sexual morph: *Ascomata*** 270–290 μm high × 370–388 μm diam. (x̄ = 283 × 374 μm, *n* = 5), solitary, scattered, semi-immersed to immersed, subglobose, slightly vertically compressed, coriaceous, rough-walled, brown to dark brown with a central papillate and periphysoid ostiole. ***Peridium*** 15–40 μm (x̄ = 25 μm, *n* = 10), composed of reddish-brown to dark brown cells of *textura angularis* to *textura globulosa* becoming hyaline gelatinous towards the inner layers. ***Hamathecium*** composed of 1.5–3.5 μm (x̄ = 2.3 μm, *n* = 20) wide, numerous, dense, narrow, transversely septate, branched, anastomosing pseudoparaphyses. ***Asci*** 75–185 × 25–40 μm (x̄ = 130 × 32 μm, *n* = 20), 8-spored, bitunicate, fissitunicate, obovoid to oblong, with short a pedicel and well-developed, conspicuous ocular chamber in immature asci, less distinct at maturity. ***Ascospores*** 40–52 × 10–15 μm (x̄ = 47 × 13 μm, *n* = 20), biseriate, sometimes overlapping, broadly fusiform, 6–8-transversely euseptate, constricted at the septa, guttulate, rough-walled, with indentations present, hyaline to brown (at germination), surrounded by a 3.5–11 μm wide, mucilaginous sheath. **Asexual morph**: Not observed.

##### Culture characteristics.

Colonies on PDA reaching 50 mm diam. after 2 months at 20–25 °C, dense and circular; surface dark brown, centrally raised to form a conspicuous umbonate mound, with compact mycelium; margin irregular to lobate, papillate and fairly fluffy, covered with white aerial mycelium; reverse dark brown at the centre, becoming reddish brown to light brown towards the edge.

##### Material examined.

Thailand • Chiang Rai Province, Nang Lae, Dead stems of *Azadirachta indica* A. Juss. (*Meliaceae*), 22 June 2024, Ashani D. Madagammana, AD 0702 (MFLU 26-0215); living culture, (AD 0702) MFLUCC 26-0210.

##### GenBank accession numbers.

MFLUCC 26-0210: ITS: PZ445651, LSU: PZ456343, SSU: PZ465542, *tef*1-α: PZ495686.

##### Notes.

Based on the multi-locus phylogenetic analysis, our strain MFLUCC 26-0210 grouped with the ex-type strain (MFLU 17-0002) and another isolate (MFLU 17-0559) of *Neoaquastroma bauhiniae* with 100% ML and 1.00 BPP support (Fig. 7). Our isolate is morphologically similar to the holotype of *N. bauhiniae*, which was collected from a dead twig of *Bauhinia variegata* in Phrae Province, Thailand (Phukhamsakda et al. 2018). However, our isolate has comparatively larger ascomata (270–290 × 370–388 μm vs. 113–190 × 170–307 μm), a thicker peridium (15–40 μm vs. 8–25 μm), and longer asci (75–185 μm vs. 53–116 μm) than the holotype of *N. bauhiniae* (Phukhamsakda et al. 2018). The fungal strain MFLUCC 26-0210 was identified as *N. bauhiniae* based on morphological and phylogenetic analyses. Previously, this species was reported only on *Bauhinia variegata* and *Bauhinia purpurea* (*Fabaceae*) hosts in Thailand. In this study, a new host record on *Azadirachta indica* from Thailand is also reported.

#### Pararoussoella

Taxon classificationFungiPleosporalesThyridariaceae

Wanas., E.B.G. Jones & K.D. Hyde

FDB586F1-2C7E-5ABD-B541-70A28AEF5D2C

##### Notes.

Based on morphology and molecular data, Wanasinghe et al. (2018) introduced *Pararoussoella* under *Thyridariaceae*, with *P. rosarum*, collected from *Rosa* sp. in the United Kingdom, as the type species. Later, Phookamsak et al. (2019) listed *Pararoussoella* under *Roussoellaceae* based on phylogenetic analysis of LSU, SSU, ITS, *tef*1-α, and *rpb*2 sequence data with broad taxon sampling. This genus was described based on the sexual morph, characterised by immersed, globose, ostiolate ascomata; filamentous, branched pseudoparaphyses; cylindrical, bitunicate asci; and narrowly ellipsoid, guttulate ascospores with irregular longitudinal striations (Wanasinghe et al. 2018). Several species with asexual morphs have also been reported, such as *P. juglandicola*, *P. lincangensis*, *P. coffeae*, *P. quercina*, and *Pararoussoella hydei* (Crous et al. 2019; Crous et al. 2020; Ren et al. 2024; Lu et al. 2025; Xiao et al. 2025). Currently, Species Fungorum (2026) lists eight species in this genus, which have been reported in several countries, including the UK, China, Germany, Thailand, and Ukraine. A new host record of *P. lincangensis* on *Azadirachta indica* in Thailand is reported here.

#### Pararoussoella lincangensis

Taxon classificationFungiPleosporalesThyridariaceae

G.C. Ren & K.D. Hyde, in Ren, Jayasiri, Tibpromma, Farias, Chethana, Faraj, Wanasinghe, Xu, Hyde & Gui, Mycosphere 15(1): 1044 (2024)

042CA78B-4D12-5EF8-9AC9-5D95E3A35601

Index Fungorum: IF901359

Facesoffungi Number: FoF13893

Fig. 10

##### Type.

China • Yunnan Province, Lincang, on dead woody twigs of *Heliciopsis terminalis* (*Proteaceae*), 12 August 2020, G.C. Ren, LC55 (holotype HKAS 122756); ex-type cultures KUMCC 21-0619 and KUMCC 21-0620.

**Figure 10.**
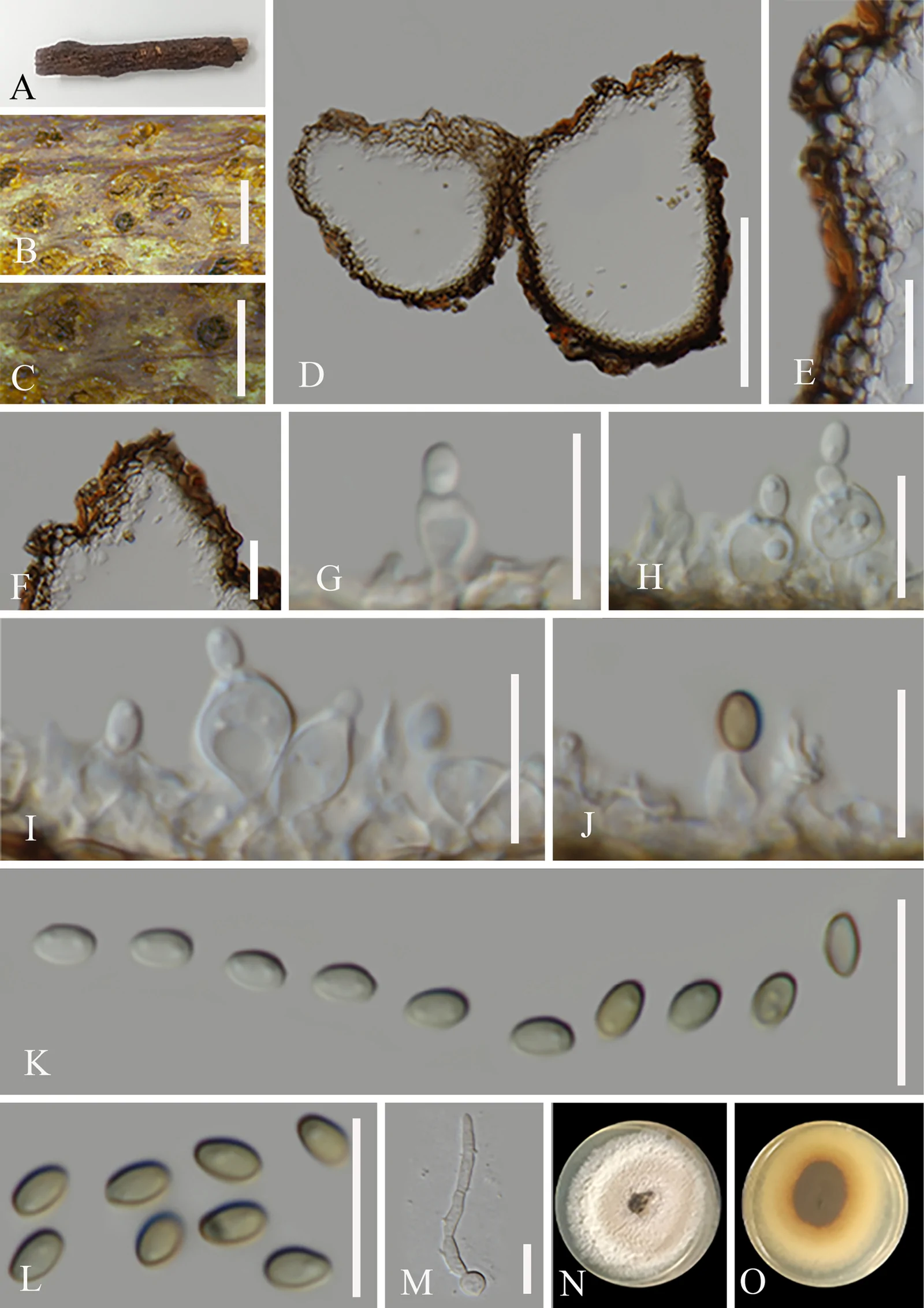
*Pararoussoella lincangensis* (MFLU 26-0216). **A**. Dead stems of *Azadirachta indica* (neem); **B, C**. Appearance of conidiomata on host substrate; **D**. Section of a conidioma; **E**. Section of conidiomatal wall; **F**. Ostiole; **G–J**. Conidiogenous cells and conidia; **K, L**. Conidia; **M**. A germinated conidium; **N, O**. Fungal colony on PDA (upper: **N**; lower: **O**). Scale bars: 2.5 mm (**B, C**); 100 μm (**D**); 20 μm (**E, F, M**); 10 μm (**G–L**).

##### Description.

***Saprobic*** on dead stems of *Azadirachta indica*. **Sexual morph**: Undetermined. **Asexual morph**: Coelomycetous. ***Conidiomata*** 130–190 µm high × 100–160 µm diam. (x̄ = 170 × 130 µm, *n* = 5), mostly solitary, scattered, erumpent through the substrate, globose to mainly sub-globose, unilocular, brown to dark brown, coriaceous, with a central, papillate ostiole. ***Conidiomatal wall*** 6–15 µm wide (x̄ = 11 μm, *n* = 20), 2–5-layered, *textura angularis* cells, outer layer cells dark brown, thick-walled, inner layers hyaline to sub-hyaline. ***Conidiophores*** reduced to conidiogenous cells. ***Conidiogenous cells*** 4–8 × 3–6 μm (x̄ = 5.9 × 4.5 μm, *n* = 20), enteroblastic, phialidic, discrete, hyaline, smooth-walled, ampulliform to doliiform, with a short, narrow neck, producing solitary conidia at the apex. ***Conidia*** 3–4.5 × 1.9–2.6 μm (x̄ = 3.7 × 2.2 μm, *n* = 30 *L/W* = 1.7), initially hyaline, becoming olivaceous at maturity even when attaching to the conidiogenous cells, smooth and thick-walled, ellipsoidal to ovoid, aseptate, with 1–2 small guttules and rounded ends.

##### Culture characteristics.

Colonies on PDA reaching 40 mm diam. after 14 days at 20–25 °C. Colonies from above white at the margin, cream at the centre, superficial, circular, flat, umbonate, with an entire edge; reverse dark greyish brown to olivaceous brown, surrounded by a deep amber to golden brown ring and fading outward to pale yellow to cream towards the margin.

##### Material examined.

Thailand • Chiang Rai Province, Nang Lae, Dead stems of *Azadirachta indica* A. Juss. (*Meliaceae*), 14 October 2024, Ashani D. Madagammana, AD 0703 (MFLU 26-0216); living culture, (AD 0703) MFLUCC 26-0211.

##### GenBank accession numbers.

MFLUCC 26-0211: ITS: PZ445652, LSU: PZ456344, *tef*1-α: PZ495687.

##### Notes.

Our strain MFLUCC 26-0211 clustered with the ex-type strain (KUMCC 21-0619), other strains (KUMCC 21-0620, MFLUCC 24-0532, MFLUCC 24-088, and MFLUCC 24-089) of *Pararoussoella lincangensis* as well as two strains of *Pararoussoella* sp. (UESTCC:23.0423 and UESTCC:23.0422), in the phylogenetic analysis and formed a well-supported clade (100% ML and 1.00 BPP), sister to *Pararoussoella hydei* (KUNCC 23-14357) (Fig. 9). Our isolate is morphologically similar to the holotype of *P. lincangensis*, which was collected from dead twigs of *Heliciopsis terminalis* (*Proteaceae*) in Yunnan Province, China. The strain MFLUCC 26-0211 was identified as *P. lincangensis* based on similar morphological characteristics and phylogenetic analyses. Previously, this species was reported from *Heliciopsis terminalis* in China, dead wood of *Dalbergiacana (Fabaceae)* in Thailand, and forest soil in Thailand (Ren et al. 2024; Afshari et al. 2025; Yasanthika et al. 2025). In this study, a new host record of *Azadirachta indica* from Thailand is reported.

### Global checklist of microfungi in *Azadirachta*

Suppl. material 1 provides a global checklist of microfungi associated with *Azadirachta*, comprising 212 fungal species. *Azadirachta* is currently represented by two accepted species, *A. indica* and *A. excelsa*, according to Plants of the World Online (POWO 2026). The checklist includes 210 fungal species from *A. indica*, one from *A. excelsa*, and one from *Azadirachta* sp., based on records in the published literature. The checklist includes information on the host species, recorded host part or substrate, country, life mode, and relevant references.

### Taxonomic distribution of microfungi associated with *Azadirachta indica*

Due to the vast majority of fungal records being associated with *Azadirachta indica*, taxonomic distribution analyses at the family and order levels were conducted only for species reported from *A. indica*.

#### Family-level fungal distribution

**Figure 11.**
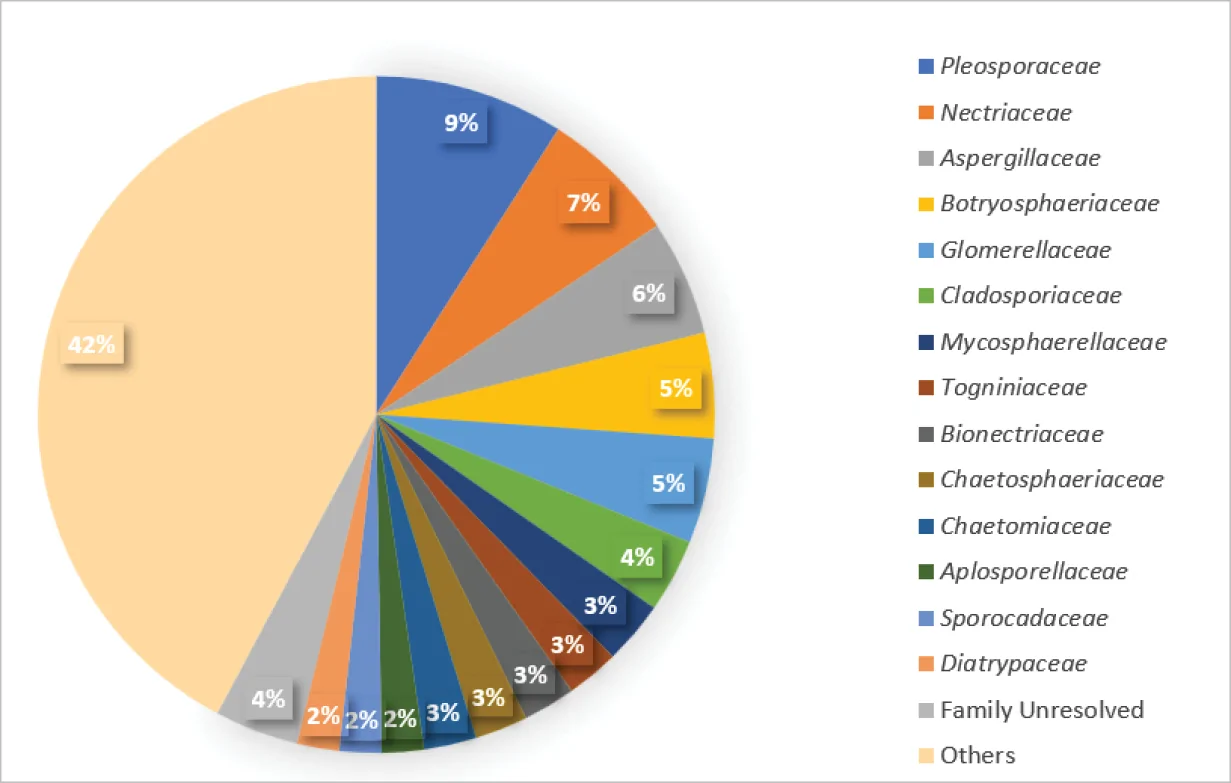
Family-level distribution of fungal species in *Azadirachta indica*.

Based on the checklist, microfungal species recorded on *Azadirachta indica*, excluding those with uncertain taxonomic status or unresolved nomenclatural placement, belong to 73 fungal families, while eight species remain unresolved at the family level. The family-level distribution of fungal species from *A. indica* is presented in Fig. 11. Families represented by more than three species were listed individually, whereas those with three or fewer species were combined under “Others” for visual clarity. Unresolved taxa are presented separately as “Family unresolved”. Based on the checklist and the chart of family-level species distribution reported from *Azadirachta indica*, *Pleosporaceae* was the most common family, with 18 species, followed by *Nectriaceae* (13), *Aspergillaceae* (11), *Botryosphaeriaceae* (10), and *Glomerellaceae* (10). Among other *Azadirachta* species, only *A. excelsa* has been recorded as hosting a member of *Nectriaceae* (*Neocosmospora keratoplastica*). Additionally, *Pseudocercospora subsessilis*, reported from *Azadirachta* sp. (without an accurate host species name), belongs to *Mycosphaerellaceae*.

#### Order-level fungal distribution

**Figure 12.**
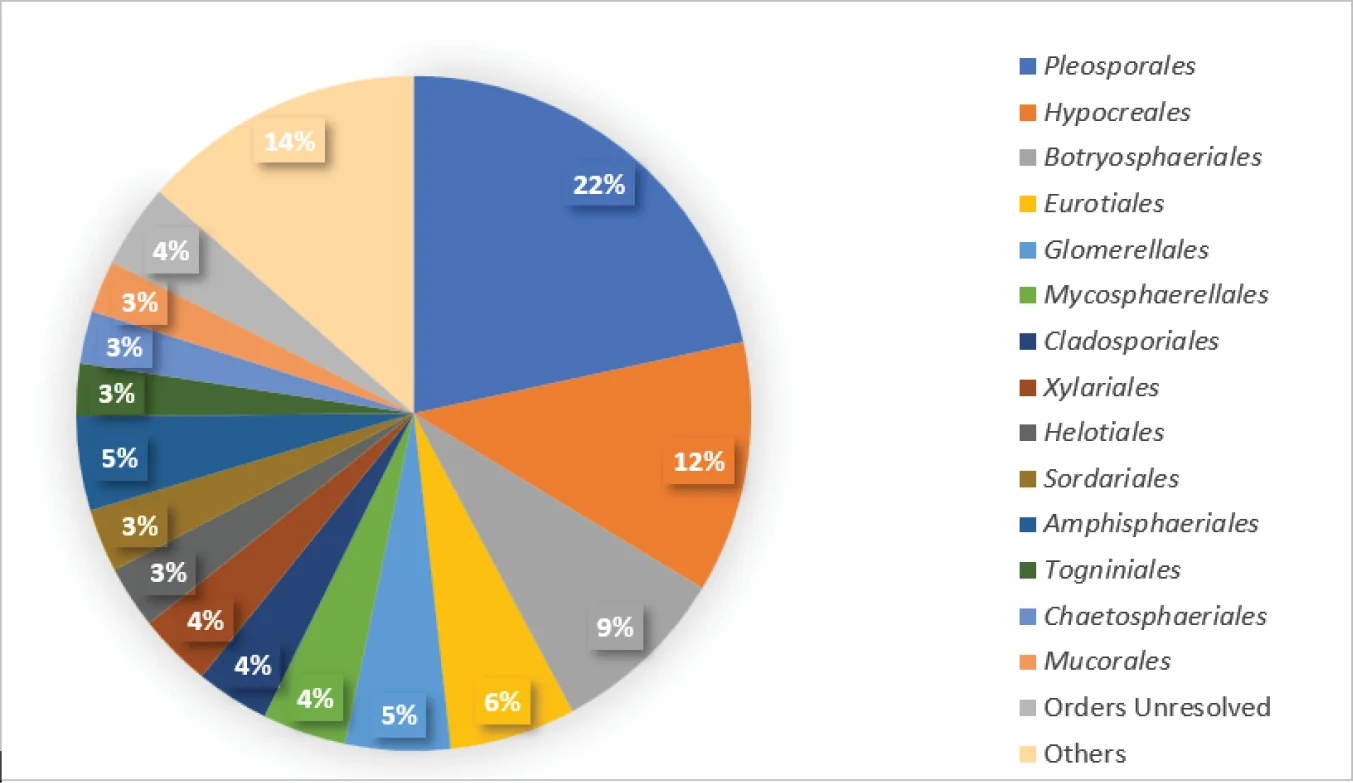
Order-level distribution of fungal species in *Azadirachta indica*.

The species associated with *A. indica* (excluding those with uncertain taxonomic status or unresolved nomenclatural placement) are distributed across 31 fungal orders, with eight species unresolved at the order level. The order-level distribution of fungal species from *A. indica* is presented in Fig. 12. Orders represented by more than four species were listed individually, whereas orders with four or fewer species were combined and listed under “Others”. Unresolved taxa are presented separately as “Order unresolved”. Based on the checklist and the chart of order-level distribution of fungal species reported from *Azadirachta indica*, *Pleosporales* was identified as the most common order, with 43 species, followed by *Hypocreales* (24), *Botryosphaeriales* (17), *Eurotiales* (12), and *Glomerellales* (10). Among other *Azadirachta* species, *Pseudocercospora subsessilis*, reported from *Azadirachta* sp. (without a clearly identified host species), belongs to *Mycosphaerellales*. In addition, the only recorded species from *A. excelsa* (*Neocosmospora keratoplastica*) belongs to *Hypocreales*.

### Ecological and geographical patterns of microfungi associated with *Azadirachta*

**Figure 13.**
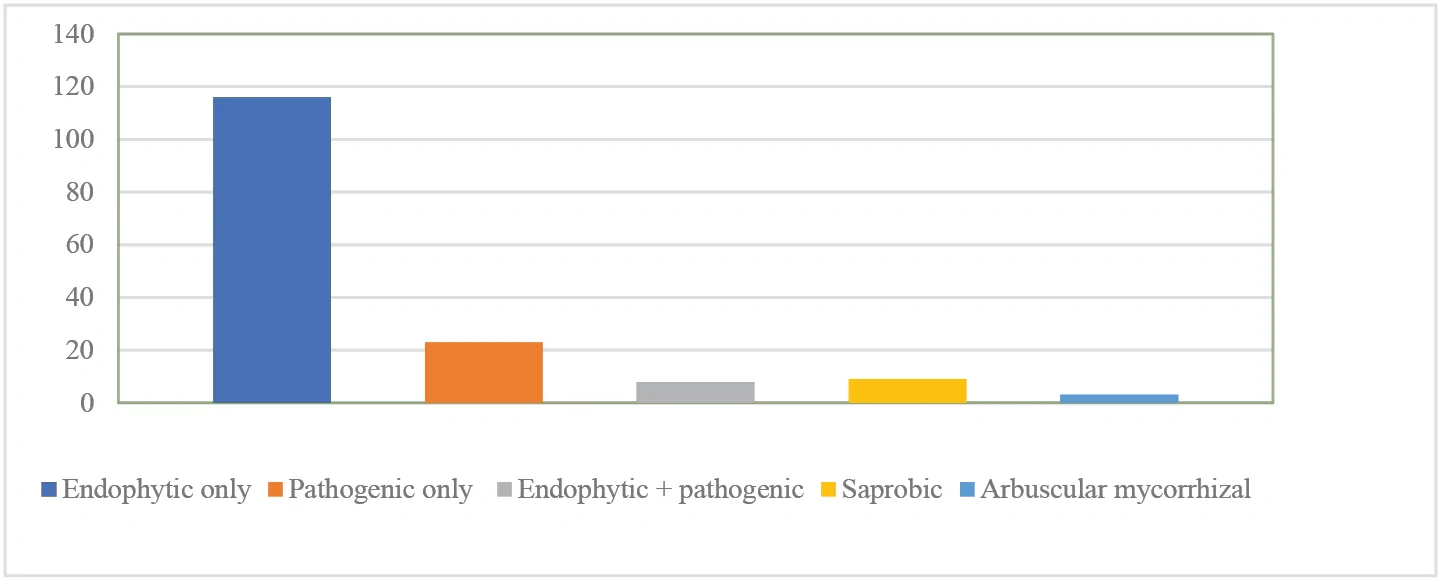
Life mode distribution of microfungal species in *Azadirachta indica*.

According to the literature, most fungal species associated with *Azadirachta* were reported as endophytes (124 species), followed by pathogens (31 species), saprobes (nine species), and arbuscular mycorrhizal fungi (three species) (Fig. 13). Among the reported life modes of fungi associated with *Azadirachta* hosts, eight species were reported as both endophytes and pathogens. However, based on the available literature, the checklist includes 53 species without a reported life mode.

*Azadirachta indica*-associated microfungi were reported from 18 countries. India had the highest number of distinct species (105), followed by China (45), Pakistan (25), Oman (24), Nigeria (18), Brazil (14), and Iran (11). Thailand had five recorded species, all documented in the present study, whereas seven countries had only one recorded species each.

## Discussion

Thailand lies within a global biodiversity hotspot called the Greater Mekong Subregion (GMS) and is considered to have a high diversity of both macro- and microfungi (Chethana et al. 2023; Hyde et al. 2023; Mukhopadhyay et al. 2024). In this study, microfungi associated with *Azadirachta indica* were collected in northern Thailand, a region reported to have a species novelty rate ranging from 55% to 96% (Hyde et al. 2018). Hyde and co-workers have studied fungal diversity in northern Thailand for nearly two decades, with ongoing research documenting numerous micro- and macrofungi and their associations with various hosts (Phukhamsakda et al. 2020, 2022; Bhunjun et al. 2022; Jayawardena et al. 2022; Hyde et al. 2024a). Despite nearly two decades of intensive exploration, fungal diversity in northern Thailand remains incompletely documented, with new taxa still being reported from the region (Hyde et al. 2018; Bhunjun et al. 2019; Afshari et al. 2025; Win et al. 2025). However, some host plants, including *Azadirachta indica*, remain comparatively understudied.

In this study, saprobic microfungal species were collected and isolated from *Azadirachta indica* woody litter in Chiang Rai Province (northern Thailand), including both sexual and asexual states. Based on morphology and multi-locus phylogenetic analyses, five *Pleosporales* species were identified, including two new species (*Neomassaria azadirachtae* and *Nigrograna azadirachtae*) and three new host records of *Aquadictyospora yunnanensis*, *Neoaquastroma bauhiniae*, and *Pararoussoella lincangensis* from *A. indica*. Of these three species, *Aquadictyospora yunnanensis* is also a new geographical record from Thailand. Of the two newly introduced species, *Neomassaria azadirachtae* is represented by a sexual morph, whereas *Nigrograna azadirachtae* is represented by an asexual morph. However, *Nigrograna* was not treated in the monograph of hyaline-spored coelomycetes (Li et al. 2020), although several morphologically similar genera, such as *Asteromella* and *Dendrodomus*, were illustrated in that monograph but lack molecular data. *Azadirachta indica* contains several secondary metabolites with antimicrobial activity in its fruits, leaves, and wood extracts (Saleem et al. 2018; Wylie and Merrell 2022). Its chemical profile may therefore affect substrate characteristics and potentially shape saprobic fungal colonisation patterns (Birkemoe et al. 2022). Furthermore, reporting new fungal species, new host records, and geographical records of fungi is also important for the proper estimation and taxonomic classification of fungal diversity and for determining host-jumping patterns, host-shift speciation, and the adaptations of fungi during their life cycles (Hyde et al. 2020b, 2024a; Samarakoon et al. 2021; Bhunjun et al. 2022; Phukhamsakda et al. 2025).

In addition to providing novel taxonomic contributions and documenting host and geographical extensions, this global checklist of microfungi associated with *Azadirachta* highlights broader patterns in the diversity and distribution of fungal communities associated with members of this host genus. This checklist shows an uneven distribution of fungal species among different *Azadirachta* species, with 210 species recorded from *A. indica*, while *A. excelsa* has only one fungal species. This uneven pattern likely reflects sampling bias, driven by the economic and ethnomedicinal importance of *A. indica* relative to other *Azadirachta* species. Family-level and order-level distributions of recorded fungal species in *A. indica* revealed that the highest number of species belonged to *Pleosporaceae* in *Pleosporales*. This pattern may reflect the ecological versatility of species within *Pleosporales*, which are widely reported from a variety of plant hosts and substrates. In the present global checklist, the 43 *Pleosporales* species associated with *A. indica* include five species reported in this study. Further systematic sampling of *A. indica* across different geographical regions and host substrates may reveal additional pleosporalean species and new host–fungal associations. Similar taxonomic patterns have been reported in some studies of plant-associated fungi, including investigations of fungal endophytes from arid-zone plants by González-Menéndez et al. (2018). Further, Karimi et al. (2025) and Htet et al. (2025) also reported *Pleosporales* as one of the prominent orders in their fungal studies conducted in Thailand, involving palms in peat swamps and weeds in *Asteraceae*, respectively.

In the checklist, life-mode information could not be retrieved for some species (over 50) recorded from *A. indica*. Among species with available data, endophytes accounted for the highest number (124), followed by pathogens (31), saprobes (nine), and arbuscular mycorrhizal fungi (three species). This distribution of life modes likely reflects research bias, as many studies have focused on endophytic fungal communities in *Azadirachta indica* due to their rich bioactive compounds and medicinal properties (e.g. Verma et al. 2011; Qureshi et al. 2019). Regarding the geographical distribution of microfungi associated with *A. indica*, the higher number of microfungal species reported from India than from other countries may reflect the long history of research on the host and its associated fungi. In contrast, only five species were recorded from Thailand in the present study, with no previous records identified in the literature compiled for this checklist, suggesting that microfungal diversity associated with *A. indica* may be considerably understudied in Thailand.

In conclusion, this study expands the known diversity of microfungi associated with *Azadirachta indica* by describing two new species and three new host records, one of which is a new geographical record for Thailand. The worldwide checklist of microfungi in *Azadirachta* reveals substantial gaps in knowledge of *Azadirachta*-associated microfungi, particularly among less-studied host species and geographical regions. The comparatively limited number of microfungal species recorded on this host from Thailand also highlights the requirement for further studies of microfungi associated with *A. indica*. Therefore, targeted future sampling efforts with integrative taxonomic approaches are essential for addressing current sampling biases and achieving a more comprehensive estimate of fungal diversity within this genus.

## Supplementary Material

XML Treatment for Neomassaria

XML Treatment for Neomassaria azadirachtae

XML Treatment for Nigrograna

XML Treatment for Nigrograna azadirachtae

XML Treatment for Aquadictyospora

XML Treatment for Aquadictyospora yunnanensis

XML Treatment for Neoaquastroma

XML Treatment for Neoaquastroma bauhiniae

XML Treatment for Pararoussoella

XML Treatment for Pararoussoella lincangensis

## Additional information

### Artificial Intelligence (AI) use

The authors accept full responsibility for the content of the manuscript, including the disclosure of any use of AI.

No AI tools were used in the preparation of this manuscript.

### Author ORCIDs

A.D. Madagammana https://orcid.org/0009-0004-9199-0632

C.S. Bhunjun https://orcid.org/0000-0001-8098-3390

C. Phukhamsakda https://orcid.org/0000-0002-1033-937X

D.-q. Dai https://orcid.org/0000-0001-8935-8807

S. Ali https://orcid.org/0000-0002-0931-0633

S.C. Karunarathna https://orcid.org/0000-0001-7080-0781

K.D. Hyde https://orcid.org/0000-0002-2191-0762

H. Wang https://orcid.org/0000-0002-2128-7894

### Data availability

All sequence data generated in the current study are available in NCBI GenBank.

## Supplementary materials

Supplementary material 1 Worldwide checklist of fungal species associated with *Azadirachta* hosts Data type xlsx https://binary.pensoft.net/file/1809128This dataset is made available under the Open Database License (http://opendatacommons.org/licenses/odbl/1.0/). The Open Database License (ODbL) is a license agreement intended to allow users to freely share, modify, and use this Dataset while maintaining this same freedom for others, provided that the original source and author(s) are credited. Ashani D. Madagammana, Chitrabhanu S. Bhunjun, Chayanard Phukhamsakda, Dong-qin Dai, Sajad Ali, Samantha C. Karunarathna, Kevin D. Hyde, Haohan Wang
